# Offline eLearning for undergraduates in health professions: A systematic review of the impact on knowledge, skills, attitudes and satisfaction

**DOI:** 10.7189/jogh.04.010405

**Published:** 2014-06

**Authors:** Kristine Rasmussen, José Marcano Belisario, Petra A Wark, Joseph Antonio Molina, Stewart Lee Loong, Ziva Cotic, Nikos Papachristou, Eva Riboli–Sasco, Lorainne Tudor Car, Eve Marie Musulanov, Holger Kunz, Yanfeng Zhang, Pradeep Paul George, Bee Hoon Heng, Erica Lynette Wheeler, Najeeb Al Shorbaji, Igor Svab, Rifat Atun, Azeem Majeed, Josip Car

**Affiliations:** 1 Global eHealth Unit, School of Public Health, Imperial College London, London, UK; 2 National Healthcare Group, Singapore; 3 Department of Integrated Early Childhood Development, Capital Institute of Pediatrics, Chaoyang District Beijing, 100020, P.R. China; 4 Knowledge, Ethics and Research, World Health Organization, Geneva, Switzerland; 5 Department of Family Medicine, Medical Faculty, University of Ljubljana, Ljubljana, Slovenia; 6 Department of Global Health and Population, Harvard School of Public Health, Harvard, MA, USA; 7 Health Services and Outcomes Research Programme, Lee Kong Chian School of Medicine, Imperial College & Nanyang Technological University, Singapore

## Abstract

**Background:**

The world is short of 7.2 million health–care workers and this figure is growing. The shortage of teachers is even greater, which limits traditional education modes. eLearning may help overcome this training need. Offline eLearning is useful in remote and resource–limited settings with poor internet access. To inform investments in offline eLearning, we need to establish its effectiveness in terms of gaining knowledge and skills, students’ satisfaction and attitudes towards eLearning.

**Methods:**

We conducted a systematic review of offline eLearning for students enrolled in undergraduate, health–related university degrees. We included randomised controlled trials that compared offline eLearning to traditional learning or an alternative eLearning method. We searched the major bibliographic databases in August 2013 to identify articles that focused primarily on students’ knowledge, skills, satisfaction and attitudes toward eLearning, and health economic information and adverse effects as secondary outcomes. We also searched reference lists of relevant studies. Two reviewers independently extracted data from the included studies. We synthesized the findings using a thematic summary approach.

**Findings:**

Forty–nine studies, including 4955 students enrolled in undergraduate medical, dentistry, nursing, psychology, or physical therapy studies, met the inclusion criteria. Eleven of the 33 studies testing knowledge gains found significantly higher gains in the eLearning intervention groups compared to traditional learning, whereas 21 did not detect significant differences or found mixed results. One study did not test for differences. Eight studies detected significantly higher skill gains in the eLearning intervention groups, whilst the other 5 testing skill gains did not detect differences between groups. No study found offline eLearning as inferior. Generally no differences in attitudes or preference of eLearning over traditional learning were observed. No clear trends were found in the comparison of different modes of eLearning. Most of the studies were small and subject to several biases.

**Conclusions:**

Our results suggest that offline eLearning is equivalent and possibly superior to traditional learning regarding knowledge, skills, attitudes and satisfaction. Although a robust conclusion cannot be drawn due to variable quality of the evidence, these results justify further investment into offline eLearning to address the global health care workforce shortage.

The world is short of 7.2 million health–care workers and this figure is growing [1]. The shortage of teachers is even greater, which limits traditional education modes. Health workers are fundamental to ensuring equitable access to health services and achieving universal health coverage. In 2006, the World Health Organization (WHO) reported that fifty–seven countries were facing critical health workforce shortages due to lack of adequate training or migration (brain drain) [2]. Although major progress has been made to tackle the earlier estimated shortage of 4.3 million health workers globally [2], the numbers of health workers still need to be scaled up considerably [3] to achieve the Millennium Development Goals [4].

eLearning might help to address the training need for health workers. Many universities are already using eLearning to support traditional campus–based education or enable access to distance or flexible learning. Perceived advantages include reduction of the costs associated with delivery of educational outcomes [5], improving scalability of educational developments [6], increasing access and availability to education by breaking down geographical and temporal barriers and allowing access to experts and novel curricula [7].

eLearning is “an approach to teaching and learning, representing all or part of the educational model applied, that is based on the use of electronic media and devices as tools for improving access to training, communication and interaction and that facilitates the adoption of new ways of understanding and developing learning” [8]. It does not only differ from traditional learning (ie, face–to–face learning that takes place in a classroom environment) in the medium by which learning is delivered [9], but also affects the teaching and learning approaches used. eLearning can take the form of a full eLearning approach, which is entirely driven by technology, or be a mix of the traditional and fully computer–based methodologies (blended learning). Blended learning might be more suitable for health care training because of the need to combine hands–on skills–based training at practical level as well as self–directed learning [10–14].

The United Nations (UN) and the WHO regard eLearning as a useful tool in addressing education needs in health care, especially in developing countries [15,16] where the worst health workforce shortages occur [2]. Currently, the most renowned eLearning initiatives focus on the online delivery of and online interaction with the learning materials. However, in resource–limited settings this approach is often not possible. Only 31% of the population had internet access in developing countries in 2013 [17]. Because network connectivity and bandwidth availability are key obstacles to effective delivery of eLearning content [9,18,19], a partially or completely offline eLearning approach may be more suitable in rural and/or developing areas. Offline computer–based eLearning delivered through eg, a CD–ROM or USB stick, for example, can be particularly efficient in increasing the accessibility, quality and availability of health related education within limited costs in remote areas with limited teaching staff, equipment, technological infrastructures and resources available. Assessing the effectiveness of these interventions for health professional education could provide an evidence base to guide and inform future projects and policies aimed at addressing the global shortage of health workers.

To our knowledge only 2 systematic reviews of randomised controlled trials (RCTs) assessing the effectiveness of offline eLearning have been conducted so far [20,21]. Both reviews were published over a decade ago. Besides, they only focused on dentistry [21] and medical [20] education.

We conducted a systematic review to compare the effectiveness of offline eLearning with traditional learning in terms of gaining knowledge and skills, students’ satisfaction and attitudes towards eLearning.

## METHODS

We conducted a systematic review following the Cochrane methodology [22].

### Search methods for identification of studies

**Electronic searches .**We limited our electronic searches to records published on or after the year 2000 in order to highlight recent developments.

We developed a search strategy for MEDLINE (OvidSP) using a combination of keywords and MeSH terms that captured the types of intervention and the types of participants under evaluation in this systematic review (**Table 1**). We adapted the search strategy for use in EMBASE (OvidSP), PsycINFO (Ovid SP), Cochrane Central Register of Controlled Trials (CENTRAL), Web of Science, and Educational Resources Information Center (ERIC) (ProQuest).

**Table 1:** Search strategy for use in MEDLINE (Ovid SP)*

1.	exp Education, Distance/
2.	educat$.mp.
3.	learn$.mp.
4.	train$.mp.
5.	instruct$.mp.
6.	2 or 3 or 4 or 5
7.	“computer assisted”.mp.
8.	Internet.mp
9.	distance.mp.
10.	web.mp.
11.	online.mp.
12.	virtual.mp.
13.	“mobile phone”.mp.
14.	“cell$ phone”.mp.
15.	smartphone
16.	smart–phone
17.	7 or 8 or 9 or 10 or 11 or 12 or 13 or 14 or 15 or 16
18.	6 adj3 17
19.	exp Computer–Assisted Instruction/
20.	eLearning.mp.
21.	e–Learning.mp.
22.	mLearning.mp.
23.	m–Learning.mp.
24.	“virtual learning environment”.mp.
25.	1 or 18 or 19 or 20 or 21 or 22 or 23 or 24
26.	exp Education, Medical, Undergraduate/
27.	exp Education, Nursing/
28.	exp Medical Staff/
29.	exp Physicians/
30.	doctor?.mp.
31.	physician?.mp.
32.	exp Physician Assistants/
33.	exp Nurses/
34.	nurse?.mp/
35.	exp Nurses’ Aides/
36.	exp Allied Health Personnel/
37.	exp Community Health Workers/
38.	exp Health Personnel/
39.	exp Health Manpower/
40.	26 or 27 or 28 or 29 or 30 or 31 or 32 or 33 or 34 or 35 or 36 or 37 or 38 or 39
41.	25 and 40
42.	Randomized controlled trial.pt.
43.	Controlled clinical trial.pt.
44.	Randomized.ab.
45.	Placebo.ab.
46.	Drug therapy.fs.
47.	Randomly.ab.
48.	Trial.ab.
49.	Groups.ab.
50.	42 or 43 or 44 or 45 or 46 or 47 or 48 or 49
51.	exp animals/ not humans.sh.
52.	50 not 51
53.	41 and 52
54.	Limit 53 to yr = ”2000 –Current”

*Source: Ovid MEDLINE® In_process& Other Non–Indexed Citations and Ovid MEDLINE® 1946 to Present. Date of search: 16 August2013 09:53. Limits: Year – 2000. Filter: Cochrane Highly Sensitive Search Strategy for identifying randomized trials in MEDLINE: sensitivity–maximizing version (2008 revision); Ovid format.

Where available, we used validated methodological filters to limit our searches to Randomised Controlled Trials (RCTs) and cluster RCTs (cRCTs). We ran the searches in August 2013.

**Searching other resources.** We checked reference lists of the included studies and systematic reviews of the literature identified by our electronic searches for additional studies.

### Inclusion criteria

**Types of studies and participants.** We included studies published in any language on students of (i) undergraduate, health–related university degrees; or (ii) basic, health–related vocational training programmes. We defined undergraduate education or basic vocational training as any type of study leading to a qualification that: (i) is recognised by the relevant governmental or professional bodies of the country where the studies were conducted; and (ii) entitles the qualification–holder to apply for entry level positions in the health care workforce. For this reason, graduate medical education courses from the USA were included.

We considered studies on candidates for and holders of the qualifications listed in the Health Field of Education and Training of the International Standard Classification of Education (ISCED–F) [23], except studies on students of traditional and complementary medicine. We hence included students reading dental studies, medicine, nursing and midwifery, medical diagnostic and treatment technology, therapy and rehabilitation, or pharmacy. Medicine and dentistry were classified under the umbrella term *allied health professions*.

**Types of intervention.** First, we conducted a systematic mapping of the types of technologies used by the included studies to deliver the learning materials, through which we identified 6 broad categories of eLearning interventions, based on the technologies employed: (1) Offline computer–based eLearning, (2) Online and local area network–based eLearning, (3) Psychomotor skills trainer, (4) Virtual reality environments, (5) Digital game–based learning and (6) mLearning.

We allocated each included study to the category that fitted the study best (definition of these categories is available in **Online Supplementary Document(Online Supplementary Document)**).

We only included studies in which offline eLearning interventions were used to deliver the learning content, which we defined as *standalone applications where internet or intranet connections were not required for the delivery of the learning activities*. The eLearning software and interactions thus run entirely on a PC or laptop. Delivery channels of the software could be via CD–ROM or a USB memory stick. If the delivery mode of the software was based on a networked connection but the learning activities did not rely on this connection – ie, a replacement delivery channel could easily be identified with low efforts/costs, without any restrictions on original intended usage – then this is also an offline intervention.

Only studies that compared eLearning or blended learning methods to: (i) traditional learning; (ii) an alternative eLearning or blended learning method; or (iii) no interventions were eligible for inclusion. These studies could either be studies where eLearning was the sole means by which the intervention was delivered or where eLearning was part of a complex, multi–component intervention.

**Types of outcome measures.** To be eligible for inclusion, studies had to report at least 1 of the following primary or secondary outcomes:

**Primary outcomes.** Primary outcomes were: (1) Students’ knowledge, measured using any validated or non–validated instrument (eg, pre– and post–test scores, grades, perceived knowledge survey scores); (2) Students’ skills, measured using any validated or non–validated instrument (eg, pre– and post–test scores, time to perform a procedure, number of errors made whilst performing a procedure, perceived up–skilling); (3) Students’ satisfaction and attitudes towards eLearning, measured using any validated or non–validated instrument (eg, self–efficacy, satisfaction, acceptability).

**Secondary outcomes.** Secondary outcomes were: (1) Health economic properties of the interventions (eg, implementation cost, return on investment); (2) Adverse and/or unintended effects of eLearning (eg, potential feelings of depression and loneliness, dropout risks [24] and “computer anxiety” [25]).

We only considered studies to have measured students’ satisfaction and attitudes towards eLearning if they met all of the following criteria: (i) they compared the differences between intervention and control groups for these outcomes; (ii) the content of the survey questionnaires related to the teaching method (ie, eLearning method, blended learning, or traditional learning); and (iii) the adjectives used in the survey questionnaires accurately described attitudes and/or satisfaction.

### Study selection and data collection

**Figure 1:**
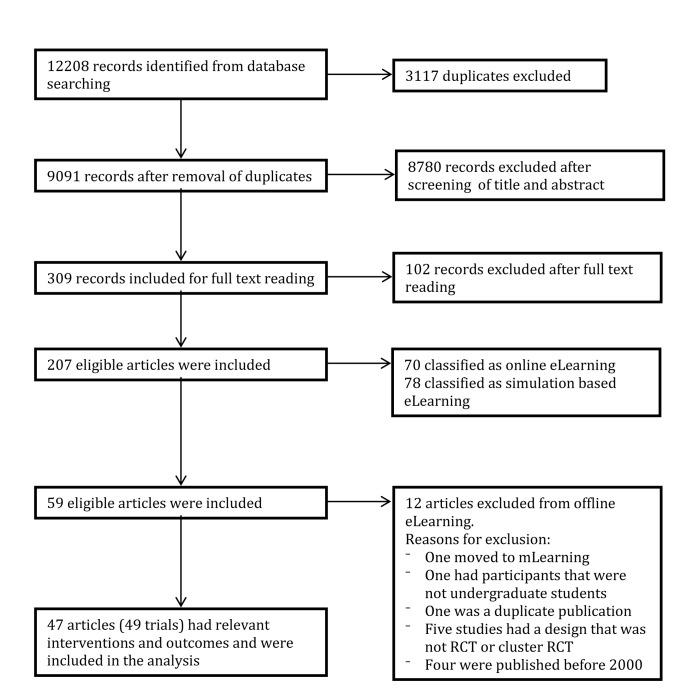
Flowchart of the studies included in the review.

The study selection process is summarised in the PRISMA flow diagram (**Figure 1**). In brief, we screened the titles and abstracts of the citations identified by our electronic and manual searches to identify potentially relevant studies, of which we assessed the full–text report to ensure they meet the inclusion criteria we specified. Review authors completed these tasks independently and met to compare their results and reach consensus.

Every selected study was allocated to a pair of review authors, with ten review authors participating in total. Each review author independently extracted data from the included studies using the structured data extraction sheet shown in **Online Supplementary Document(Online Supplementary Document)**.

Each pair of reviewers compared their completed data extraction forms and any discrepancies between review authors’ results were resolved through discussion; if no agreement could be reached, a third review author acted as an arbiter. Because ten review authors participated in the data extraction process, some categories were interpreted differently by some reviewers. Therefore, 3 reviewers went over the entire data extraction again to ensure uniformity.

We contacted authors of studies containing incomplete data to request the missing information. Some authors did not reply to our request for additional information, whilst other authors did not know the answer to our questions. For a single study, the response obtained from the author resulted in the subsequent exclusion of the study from the systematic review.

### Assessment of risk of bias in included studies

During the data extraction process, we assessed the risk of bias at the outcome level using tools recommended by the Cochrane Collaboration [22]. For RCTs, we did so across the domains of (1) random sequence generation, (2) allocation concealment, (3) blinding of participants and personnel, (4) blinding of outcome assessment, (5) incomplete outcome data, (6) selective outcome reporting, and (7) other bias including the comparability of intervention and control group; characteristics at baseline; validation of outcome assessment tools; reliability of outcome measures; and protection against contamination.

We assessed the risk of bias for cRCTs across the domains of (1) recruitment bias, (2) baseline imbalances, (3) loss of clusters and (4) incorrect analysis.

For each study, 2 reviewers independently categorised each domain as low, high or unclear risk of bias.

### Summarising the data

We qualitatively compared the characteristics of the participants and of the interventions between the included studies to determine the feasibility of conducting a meta–analysis. Because of substantial clinical, educational, content and methodological heterogeneity we did not conduct a meta–analysis. Instead, we adopted a thematic summary approach [26].

## RESULTS

The study selection process is depicted in **Figure 1**. The initial search yielded 12 208 records. After removing 3117 duplicate records using EndNote X5, we screened the titles and abstracts of 9091 records (see **Online Supplementary Document(Online Supplementary Document)** for a detailed description). After this initial screening, we excluded 8780 records. We retrieved the full text reports for the remaining 309 records and assessed them for eligibility. Of these, we excluded 102 articles that did not meet the eligibility criteria (**Figure 1**).

Forty–seven [27–73] of the remaining articles complied with the term *offline eLearning*.

Two [54,70] articles reported results of 2 separate cRCTs that were analysed separately, and 2 articles [43,74] reported results from the same study. This resulted in a total number of evaluated studies of 49 (**Table 2**).

**Table 2:** Summary of findings for the 40 studies comparing offline eLearning with traditional learning

Study	Discipline	Knowledge	Skills	Attitude	Satisfaction	No. of participants	Intervention delivery approach	Characteristics
Amesse 2008 [28]	Medicine	E				36	Full eLearning	CG: Paper based tutorial IG: Computer based tutorial
Armstrong 2009 [29]	Medicine	NS				21	Full eLearning	CG: Lecture IG: Interactive slideshow
Bains 2011 [30]	Dentistry	NS		E		90	IG 1: Full eLearning IG 2: Blended learning IG 3: Blended learning	CG: Teacher–led tutorial IG 1: Online tutorial only IG2: Online tutorial only, then teacher–led tutorial IG3: Teacher–led tutorial, then online tutorial only
Bloomfield 2010 [31]	Nursing	NS	M			223	Full eLearning	CG: Lecture/video/practice IG: Computer module including video
Boet 2010 [32]	Medicine		M			42	Blended learning	CG: Lecture IG: Lecture + CD–ROM
Bogacki 2004 [33]	Dentistry	NS				45	Full eLearning	CG: Lecture IG: Computer program
Bradley 2005 [34]	Medicine	NS		NS		168	Full eLearning	CG: Workshops IG: Workbook + CD–ROM
Davis 2008 [35]	Nursing	NS				179	Blended learning	CG: Lecture IG: Digital recording + PowerPoint slides + Internet links
Feeg 2005 [36]	Nursing	E				91	Blended learning	CG: Journal article IG: Journal article + CD
Gelb 2001 [37]	Medicine	NS				107	Full eLearning	CG: Printed tutorial IG: Computer tutorial
Glicksman 2009 [38]	Medicine		E	E		47	Full eLearning	CG: Article IG: Computer module with article
Goldsworthy 2006 [39]	Nursing	E				25	Full eLearning	CG: Paper–resources IG: PDA–based resources
Green 2011 [40]	Medicine	E			E	121	Full eLearning	CG: Paper–based resources IG: Computer program
Holt 2001 [41]	Medicine	NS				108	Full eLearning	CG: Lectures IG: Computer–based lectures
Howerton 2002 [42]	Dentistry	NS				59	Blended learning	CG: Lectures IG: CD–ROM
Jeffries 2003 [45]	Nursing	NS	NS	NS	NS	73	Full eLearning	CG: Self–study module + instructor led demonstration IG: Self–study module + CD
Kim 2003 [48]	Nursing	NS	NS	NS	E	75	Blended learning	CG: Printed material IG: Computer–based material
Kong 2009 [49]	Medicine	E	E		E	90	IG 1: Blended learning IG 2: Other learning	CG: Didactic teaching IG 1: Paper–based Problem Based Learning IG 2: Computer–based Problem Based Learning
Kurihara 2004 [50]	Medicine	E	E			59	IG 1: Full eLearning IG 2: Blended learning IG 3: Traditional learning	CG: Textbook only IG 1: Computer program only IG 2: Textbook + Computer program IG 3: No intervention
Lira 2013 [51]	Medicine	M				68	Blended learning	CG: Lecture IG: Lecture + PDF article
Maleck 2001^*^ [52]	Medicine	DNT		M	T	192	IG 1: Full eLearning IG 2: Full eLearning	CG: Paper cases, textbook + optional lecture IG 1: Computer–based cases, textbook + optional lecture IG 2: No cases, optional textbook + lecture
McDonough 2002 [53]	Medicine	NS			T	37	Blended learning	CG: Lecture + tutorial IG: Lecture + computer tutorial
McMullan 2011a^†^ [54]	Nursing	E		NS	E	48	Full eLearning	CG: Paper hand–out IG: Computer program
McMullan 2011b [54]	Nursing	E		E		50	Full eLearning	CG: Paper hand–out IG: Computer program
Miedzybrodzka 2001 [55]	Medicine	NS		NS		48	Full eLearning	CG: Lecture IG: Computer program
Nance 2009 [57]	Dentistry		NS	E		73	Full eLearning	CG: Paper hand–out + laboratory session IG: DVD only
Nola 2005 [58]	Medicine	E				85	Full eLearning	CG: Lectures + practical sessions IG: Lectures (optional) + computerised sessions
Perfeito 2008 [60]	Medicine	NS				35	Full eLearning	CG: Lecture IG: Computer program
Qayumi 2004 [63]	Medicine	E	E			99	IG 1: Traditional learning IG 2: Full eLearning IG 3: Blended learning	CG: No intervention IG 1: Text module IG 2: Computer program IG 3: Text module + computer program
Roppolo 2011 [64]	Medicine		E			180	IG 1: Blended learning IG 2: Blended learning	CG: Instructor and video based course (cognitive) + Instructor led course (practical) IG 1: Online course (cognitive) + DVD–based course (practical) IG 2: Online course (cognitive) + Facilitator based practice (practical)
Seabra 2004 [65]	Medicine	NS				60	Full eLearning	CG: Lecture IG: Computer program
Shomaker 2002^†^ [66]	Medicine	NS			DNT	94	IG 1: Full eLearning IG 2: Blended learning	CG: Lectures, texts + slides IG 1: Computer program + texts IG 2: All of the above
Solomon 2004 [67]	Medicine	NS				29	Full eLearning	CG: Lecture IG: CD–ROM
Vichitvejpaisal 2001 [69]	Medicine	M				80	Full eLearning	CG: Textbook IG: Computer program
Vivekananda–Schmidt 2005a [70]	Medicine		E	NS		105	Full eLearning	CG: No CD–ROM IG: CD–ROM
Vivekananda–Schmidt 2005b [70]	Medicine		E	E		156	Full eLearning	CG: No CD–ROM IG: CD–ROM
Weih 2008 [71]	Medicine and Psychology	NS			E	101	Full eLearning	CG: Lecture IG: Lecture + CD–ROM
Williams 2001 [72]	Medicine	NS				163	Full eLearning	CG: Lecture IG: Computer program
Xeroulis 2007 [73]	Medicine		E			60	IG 1: Blended learning IG 2: Traditional learning IG 3: Traditional learning	CG: No intervention IG 1: Computer–based video IG 2: Concurrent feedback during practice IG 3: Summary feedback after practice

E – Results favoured computer–based eLearning over traditional learning, NS – No significant difference between eLearning and traditional learning, M – Mixed results, T – Results favoured traditional learning over computer–based eLearning, DNT – Difference not tested, CG – Control group, IG – Intervention group

*Knowledge improvement in the two eLearning groups as well as the traditional learning group, whereas the control group that received no intervention, showed minimal improvement.

†In the cRCT by McMullan 2011 [55], the results for satisfaction were pooled for the two cohorts (McMullan 2011a and McMullan 2011b) and the result presented for McMullan 2011a therefore also includes students from the McMullan 2011b cohort.

‡For students’ satisfaction, no clear trends in terms of one intervention group being superior to another.

### Included studies

The 49 included studies were either parallel RCTs or cRCTs published in peer–reviewed journals between 2001 and 2013. There were no clear trends in terms of increase in publication of offline studies in the time period investigated. Thirty–five studies [27–29,32,34,37,38,40,41,44,46,47,49–53,55,56,58–70,72,73] investigated eLearning in the field of medicine, 8 in the field of nursing [31,35,36,39,45,48,54] and 4 in the field of dentistry [30,33,42,57]. One article [71] focused on both medicine and psychology whereas another [43] focused on medicine, dentistry and physical therapy at the same time.

### Participant characteristics

The total number of participants included across all trials was 4955. The smallest study included 8 participants in the control and 8 participants in the intervention group [59]. The study with the largest control group had 177 participants [58], while the largest intervention group had 113 participants [31]. Most studies were conducted among undergraduate university students apart from 2 studies [31,36] that investigated the effect of offline eLearning for vocational training. Eleven studies that specified the age of the students. In the control groups, the mean age of participants ranged from 22.4 [30] to 30 years [35]. The mean age of participants in the control group was comparable, ranging from 21.8 [30] to 30 years [35].

### Intervention characteristics

Forty studies [27–42,45,48–55,57,58,60,63–67,69–73] compared eLearning to traditional learning and 9 studies [43,44,46,47,56,59,61,62,68] compared one mode to another mode of eLearning. The shortest duration of exposure was 20 minutes [47] and the longest was 1 year [58].

**Figure 2:**
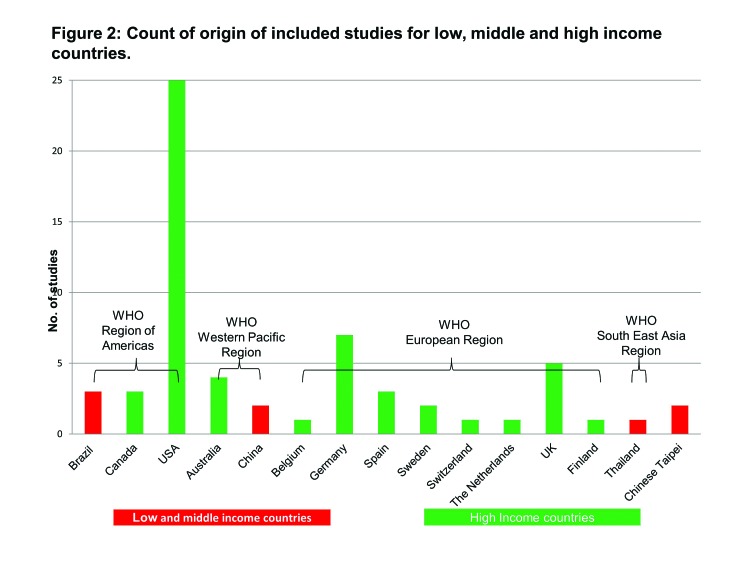
Country of origin of included, examined studies for low– and middle–income and high income countries separately

Most of the studies (42 out of 49; 86%) were conducted in high–income countries, and 13 of these [28,33,36,37,40,42,45,47,57,64,66–68] in the USA. The remaining 5 studies were conducted in low– and middle–income countries: 1 [69] in Thailand; 1 [49] in China; and 1 [51,60,65] in Brazil. **Figure 2** shows the distribution of the countries in which the studies were conducted.

The majority of the studies used programs that run on PCs or laptops [27–38,40–73]. One study [39] investigated the use of a Personal Digital Assistant (PDA), which is a small portable electronic device that can be regarded as the predecessor of a computer tablet and smartphone, with PDFs from Elsevier. Sixteen studies delivered the eLearning intervention to the students on a CD–ROM. [27,28,32–36,42,45,48,49,60,67,70,71]. The eLearning software and material used in the remaining studies were distributed via a variety of sources where specified: learning management systems such as WebCT Blackboard [30,43], DVDs [30,57,61], the internet [29,47,51], stored on a computer [53,58,69] or for 1 study [39] on a PDA, and email [56], Several interventions used standard vendor software such as Adobe® [51,54], Macromedia AuthorWare® [66] and Microsoft® PowerPoint® [29,36].

### Primary outcomes

**Students’ knowledge assessment.** Overall, 40 [27–31,33–37,39–45,47–56,58,60–63,65–69,71,72] out of the 49 studies looked at a knowledge based outcome. Nineteen of these [29–31,34,36,37,40,41,43,45,47,50,51,53,61,63,65,71,72] used only a multiple choice questionnaire (MCQ) to test students’ knowledge and understanding, while another 9 studies [27,35,44,52,55,56,58,60,66] used a MCQ in conjunction with an additional testing method (eg, short answer questions or X–ray image interpretation). A further eleven studies [28,39,42,48,49,54,62,67–69] measured students’ knowledge gain via other testing means including case analysis, X–ray image interpretation and written exams. One study [33] did not specify which method was used to examine the participants’ knowledge.

**Students’ skills assessment.** Skills were assessed in 16 studies [31,32,38,45–50,57,59,63,64,70,73], the method of which was described in all but 1 study [49]. Ten of these [31,38,45,47,48,50,63,64,70] used a rating scale and/or checklists (eg, an Objective Structured Clinical Examination – OSCE) to assess clinical skills. Three studies [46,59,73] used the Imperial College Surgical Assessment Device and a checklist for the assessment. Another study [57] used a grading rubric to assess ability to carve teeth in wax. Another study assessing the ability to conduct orotracheal fibreoptic intubation [32] evaluated successful intubation in real time.

**Students’ satisfaction and attitudes towards eLearning assessment.** None of the studies assessed change in students’ professional attitudes towards patients such as compassion.

Feedback from students assessed as their attitude towards the eLearning intervention was reported as an outcome in 14 studies [30,34,38,45,48,52,54–57,61,70]. Participants were asked to provide ratings via Likert scales in 11 studies [34,48,52,54–57,61,70]. One study [38] used a questionnaire and did not mention the use of Likert scales. In the remaining 2 studies [30,45], Likert scales were combined with another method, ie, focus groups in Bains et al. [30] and an additional questionnaire in Jeffries et al [45].

Students’ satisfaction was considered as an outcome in 13 studies [40,43,45,48,49,52–54,61,62,66,71]. Eight of these studies [40,43,49,53,54,61,62] specified that students’ satisfaction was evaluated with Likert scale questionnaires. The 5 remaining studies comparing students’ satisfaction among the students [45,48,52,66,71] used different types of questionnaires without mentioning the use of Likert scales.

### Secondary outcomes

**Health economic properties of the eLearning intervention.** Health economic properties of the eLearning intervention were rarely mentioned in the included offline eLearning studies. However, some of the studies addressed certain financial and resource related elements of eLearning. Davis et al. [35] mentioned that costs in producing the eLearning package were minimal and well within normal departmental budgets for teaching undergraduates. Ackermann et al. [27] stated that effective learning can be performed with the use of few resources and provides a very economical mode for educating medical students. Bradley et al. [34] stated that the in–house development of the eLearning course material took 40 hours for the preparation of the course material, 10 hours to administer each semester and the internet site used for the eLearning group took 100 hours to develop. The eLearning course material also included a CD–ROM produced externally with an estimated cost of £ 30 per CD [34]. McDonough et al. [53] reported that it took local IT staff 4 hours to install the program on 20 PCs and that no maintenance was required after that point. Vivekananda–Schmidt et al. [70] stated that the costs of designing the eLearning course were £ 11 740 (US$ 22 045). Tunuguntla et al. [68] wrote in reference to comparing 2 different types of eLearning: “The cost ratio (measured in hours) for the module was about 2:3: about 72h for creation of the static graphics vs. 106h for the animations”.

**Adverse or unintended effects of eLearning.** Adverse or unintended events of the eLearning intervention were not reported in any of the studies.

### Excluded studies

Initially 59 articles were categorised as offline eLearning studies. One study [75] was reclassified as mLearning because lectures were viewed on an iPod [75], and was therefore excluded from this systematic review. Eleven studies [74,76–84] were excluded during the data extraction phase because they met 1 or more of the exclusion criteria. Four studies [77,78,83,85] of these were published before 2000. Five studies [79–82,84] were excluded because the study design was not a parallel or cRCT. One study was excluded as the participants were not undergraduate students [76]. An additional study [74] was a secondary publication of a study that was already included [43] and information from the secondary publication was merged with the included study.

### Risk of bias in included studies

**Figure 3:**
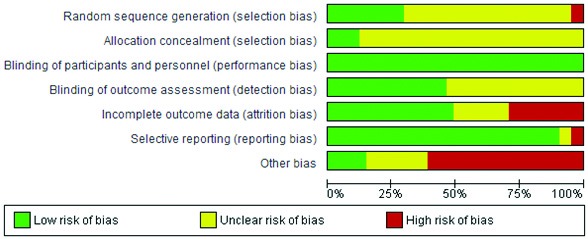
Overall risk of bias graph.

**Figure 4:**
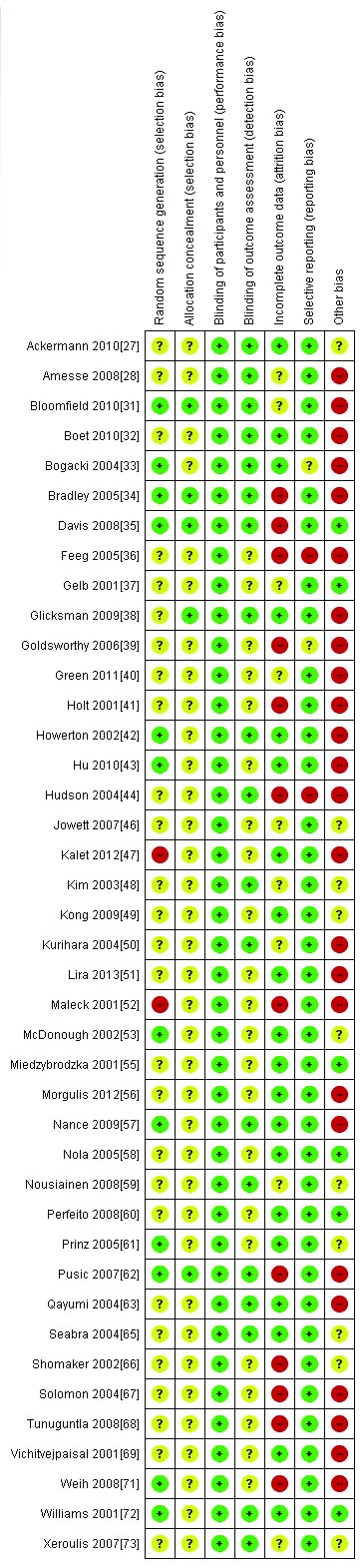
Risk of bias for each individual parallel randomised controlled trial (RCT) separately.

The assessment of risk of bias is described in detail in **Online Supplementary Document(Online Supplementary Document)**. In summary, the majority of the included parallel RCTs were considered to be of low quality because of high risk of bias [28,31–34,36,38–44,47,50–52,56,57,62,63,66–69,71]. Only a few studies [27,37,46,48,49,53,55,58–61,65,66,72,73] were of high quality with none of the assessed categories rated as having a high risk of bias (**Figure 3**** and ****4**). In the majority of studies at least 1 or more categories were classified as having an unclear risk of bias, especially with regards to the allocation of participants to intervention groups.

### Effects of offline eLearning interventions

The 49 randomized trials included in our review assessed the effectiveness of offline eLearning interventions in terms of knowledge, skills, attitudes and satisfaction. The findings were based on comparisons between offline eLearning and traditional learning or between various modes of offline eLearning. A study may have compared more than 1 outcome between groups, and each outcome may have been assessed in multiple ways. For example, a study which compared students’ acquisition of skills may have assessed skills in terms of the student’s performance on a global rating scale, ability to perform a specific procedure as well as the ability to comply with requirements in a checklist. As a result, the number of comparisons made across studies for a particular outcome may exceed the number of studies which reported that outcome.

The studies were split into 2 research themes evaluating the impact of eLearning interventions for undergraduate health care education: traditional learning vs offline eLearning, and offline eLearning vs offline eLearning.

### Traditional learning vs offline eLearning

Forty (82%) of the included studies [27–42,45,48–55,57,58,60,63–67,69–73] compared offline eLearning with traditional learning. Please refer to Table 2 for a summary of findings of the individual studies, and to **Online Supplementary Document** for a further description of the nature of the interventions.

**Students’ knowledge.** Amongst the 40 studies which compared offline eLearning with traditional learning, knowledge was assessed in 33 (83%) studies [27–31,33–37,39–42,45,48–55,58,60,63,65–67,69,72], 5 of which were cRCTs [29,30,45,54]. Eleven (33%) studies [27,28,36,39,40,49,50,54,63] assessing knowledge gain demonstrated significantly higher knowledge gains for students assigned to offline eLearning compared to those exposed to traditional learning. Outcome measures for these studies were based on correct responses to questions which included true–false, multiple choice or fill in the blanks type of assessments. The sample size for these studies ranged from 19 to 225 with all but 4 studies [36,39,54] conducted on medical students. Seven of these studies used solely offline eLearning as the main intervention [27,28,39,40,54,63,84] whereas 4 used blended learning [36,42,49,58].

None of the included studies found greater gain in knowledge for the traditional learning group.

Post–intervention knowledge was not significantly different between eLearning and traditional learning in 19(58%) of the included studies [29–31,33–35,37,41,42,45,48,53,55,60,65–67,71,72].

Two (6%) studies [51,69] showed mixed results ie, favouring the intervention, control, or neither 1 depending on the specific indicator of knowledge being assessed. Another study [51] initially found no difference between the traditional and offline eLearning groups, but statistically significantly better post–test scores were seen in the offline eLearning group after 1 month . Another study [69] showed that students taught blood gas interpretation using a textbook had greater improvement from pre–test to post–test compared to those in the offline eLearning group, but after 3 weeks the final test scores of both groups failed to show a significant difference between the 2 groups.

In 1 (3%) study [52] knowledge was assessed, but not tested for statistically significant differences between the intervention groups. The study showed knowledge improvement in the 2 offline eLearning groups as well as the traditional learning group, whereas the control group that received no intervention showed minimal improvement.

**Students’ skills.** Overall, 13 studies – 9 RCTs [31,32,38,48–50,57,63,73] and 4 cRCTs [45,64,70] measured skills as an outcome.

Of the studies that evaluated differences in skills acquisition, 8 (62%) [38,49,50,63,64,70,73] found significantly greater skills acquisition amongst students assigned to offline eLearning compared to those assigned to traditional learning. The range of skills assessed by these studies included performance in specific tasks, such as cardiopulmonary resuscitation, fibreoptic intubation and knot tying skills; performance in objective structured clinical examination, as well self–efficacy assessments. The number of participants included in these studies ranged from 19 to 354. All 8 studies [38,49,50,63,64,70,73] were conducted in medical students. Three (23%) studies [45,48,57] did not detect a significant difference in skill acquisition between groups.

None of the 13 studies demonstrated more favourable results for traditional learning compared to offline eLearning.

Results were mixed for 2 (15%) studies [31,32]. In 1 of these [31], testing hand washing skills of nursing students assigned to computer assisted vs conventional learning, skills were similar in both groups at the 2–week follow–up but were in favour of the intervention group at the eight–week follow–up. In the other study [32] that focused on intubation skills, successful intubation was more common in the offline eLearning group compared to the traditional group whereas there was no statistical significant difference in the checklist and global rating scale assessment of intubation skills.

**Students’ satisfaction and attitudes towards eLearning.** Twelve studies – 6 RCTs [34,38,48,52,55,57] and 6 cRCTs [30,45,54,70] – assessed attitudes towards the eLearning the intervention, primarily through Likert scale surveys.

Five (42%) studies [30,38,54,57,70] found more favourable results for students assigned to eLearning compared to traditional learning.

Six (50%) studies [34,45,48,54,55,70] did not detect a statistically significant difference in attitudes toward eLearning between groups. None of the studies found more favourable attitudes towards traditional learning.

One study [52] that assessed the difference between traditional learning and 2 different types of eLearning (8%) showed mixed results. The comparison between the traditional learning group and the eLearning group with no interaction (ie, offline eLearning cases with no tests) showed that statistically significantly more students would recommend eLearning group with no interaction. However, the comparison between the control and the eLearning group with interaction (ie, cases with multiple choice and free–text questions) did not show a statistically significant difference [52].

Students’ satisfaction was assessed in 7 RCT studies [40,48,49,52,53,66,71] and 2 cRCT studies [45,54].

Out of 9 studies looking at the level of students’ satisfaction, 5 (56%) studies [40,48,49,54,71] found a significantly greater proportion of students who were satisfied among those exposed to eLearning as compared to those exposed to traditional learning. Students’ satisfaction was based on questionnaires, surveys and global perceptions of satisfaction.

Two of the studies [52,53] showed higher satisfaction levels for students assigned to traditional learning groups.

One (11%) study [45] did not detect any significant difference while another study (11%) [66] did not test for significant differences and there were no clear trends in terms of 1 intervention group being superior to another.

### Comparison of different types of offline eLearning against each other

Nine (18%) [43,44,46,47,56,59,61,62,68] of the included studies compared the effectiveness of various modes of offline eLearning against each other.

**Students’ knowledge.** Seven (78%) studies [43,44,47,56,61,62,68] compared various forms of offline eLearning and their effects on knowledge. A study [43] comparing the effectiveness of 3D vs 2D images of the larynx projected on a computer screen demonstrated higher test scores for students assigned to view 2D images. Another study [61] assessing the effectiveness of an actual video of ophthalmic procedures vs actual video supplemented with 3D video demonstrated higher scores on theoretical knowledge for the group assigned to 3D video.

One study [56] comparing 2 types of eLearning for teaching a module on leukaemia found that the more interactive eLearning intervention including questions resulted in statistically significantly higher mean percentage scores on the post–test on leukaemia compared to the more passive intervention group who only saw text and had no questions to answer.

No differences were found in 3 studies [44,47,68] comparing different eLearning modalities with each other. Two of the studies [44,47] compared groups of eLearning with different levels of student interaction with each other, whereas 1 group received no intervention. The third study [68] compared the effects of 2 versions of a program, 1 with animations and 1 with static graphics.

One study [62] showed mixed findings, with 1 offline eLearning mode exhibiting superior results with respect to a particular knowledge test and another offline eLearning mode exhibiting better results with respect to a different knowledge test.

**Students’ skills.** Skill acquisition was assessed in 3 (33%) [46,47,59] of the 9 studies which compared different offline eLearning modalities. Out of the 3 studies which assessed skill, 1 study [47] demonstrated better skill acquisition with the use of a particular mode of offline eLearning over other modes. That study investigated the effects of 3 different methods of manipulating contents for learning abdominal examination: click, watch and drag. Their results showed that students who were able to use the mouse to trigger animated demonstrations (‘click’) performed better in auscultation than those who were in a more passive learning group where students only had control over the pace of the presentation (‘watch’). The same group (‘click’) outperformed students who were in a more active learning group where students were able to drag tools in motions simulating actual performance of the task (‘drag’) in terms of abdominal palpation and additional maneuvers. In addition, more students in the ‘drag’ and ‘click’ groups correctly diagnosed a simulated patient as having appendicitis than students in the ‘watch’ group.

Two studies [46,59] failed to demonstrate any difference in skill acquisition between eLearning modes.

**Students’ satisfaction and attitudes towards eLearning.** Prinz et al. [61] and Morgulis et al. [56] were the only studies amongst the 9 studies comparing different eLearning modalities that assessed attitudes towards eLearning. The study by Prinz et al. showed that the students in the 3D group rated the learning aid in the 3D group more useful compared to the control group students’ rating of the learning aid available in the control group and the difference was statistically significant. Intelligibility for glaucoma surgery and improvement of spatial ability both received statistically significantly more positive responses in the 3D group compared to the control group. However, no difference was found for intelligibility for cataract surgery [61]. Similarly, the study by Morgulis et al. [56] that compared the use of existing online resources with a purpose–built, targeted eLearning module on leukaemia for medical students demonstrated an overwhelmingly positive response from students assigned to the targeted module.

Three (33%) studies [43,61,62] compared the effects of different eLearning modes on students’ satisfaction. The study by Prinz et al. [61], earlier cited for favourable results of 3D over 2D learning of ophthalmic procedures on knowledge, reported greater student satisfaction with the 3D video. Although Hu et al.’s study [43] found that knowledge gain was higher for the 2D vs 3D learning group, enjoyment was higher in students assigned to 3D computer models. A study [62] which compared the effectiveness of a linear vs branched format for computer tutorials demonstrated that while the layout did not make a difference to their gain in ability, students in the linear group were slightly less likely to rate the tutorial as “valuable.”

## DISCUSSION

Our findings suggest that offline eLearning is at least equivalent, possibly superior to traditional learning in terms of students’ knowledge, skills, and satisfaction and attitudes towards eLearning. Unfortunately, no studies evaluated impact on learners’ professional attitudes towards patients. Eleven of the 33 studies testing knowledge gains found significantly higher gains in the eLearning intervention groups compared to traditional learning, whereas 21 did not detect significant differences or found mixed results. The remaining study did not test for differences. Eight of the 13 studies testing skill gains detected significantly higher gains in students allocated to the eLearning intervention, whilst 5 of the studies did not find statistically significant differences between the intervention and control group. Generally no differences in attitudes or preference of eLearning over traditional learning were observed, nor between different modes of offline eLearning.

Studies varied considerably in terms of type of eLearning (ie, full eLearning vs blended learning), the content, delivery channels, duration and frequency of exposure to the intervention, measures of outcomes, type of degrees, and seniority of students. For this reason, we did not calculate overall summary effect estimates. The majority of studies focused on full offline eLearning, whereas blended learning was used in fewer studies. Although the majority of studies comparing offline eLearning with traditional learning focused on seemingly similar offline eLearning programs, the extent of interaction they provided varied from a simple PDF file [51,54] on a PDA as a learning aid when learning how to do drug calculations [39] to software with quizzes and other interactive features [47]. The duration of exposure to the eLearning interventions and the time from completion of the eLearning intervention until knowledge or skills were measured ranged from 12 minutes [46] to 1 year [58]. The complexity of the eLearning modalities also varied. However, apart from 1 study that used a PDA with software that could function without the internet [39] all studies used computers.

The participants of the identified trials are representative of the intended population of students enrolled in undergraduate, health–related university degrees, and we expect that our results also apply to other similar university degrees. However, only 5 [49,51,60,65,69] of the 49 included studies were conducted in low– to middle–income countries, none of which in the Mediterranean and African regions. Because we focused on offline eLearning that does not require internet access, the limited availability of data from developing countries does not limit the scope of the review in terms of the technology studied. However, due to the fact that computer literacy and cultural factors may determine the overall effects of eLearning on all domains we studied, it is possible that our conclusions on effectiveness are not applicable to all countries and settings.

Over 50% of the studies [28,31,33,34,38,41–44,47,56,57,62,63,67–69,71,75] asked students whether they would be keen to participate in a trial on eLearning. The resulting study participants are thus likely to be more eager to use the eLearning interventions, which might have resulted in more favorable assessment of this educational approach. Indeed, among the studies showing positive effects of eLearning, 4 studies [28,38,63,64] had a high risk and 6 studies [27,36,39,40,50,73] had an unclear risk of volunteer bias.

Our results are in line with the majority of the existing literature. A review on online eLearning that we prepared in parallel also showed that the effects of online eLearning were equivalent, possibly superior to traditional learning. Likewise, a systematic review of 12 RCTs on computer–aided learning in dental education including both undergraduate students and dentists reported that statistically significant differences were not detected in the majority of studies comparing eLearning and traditional learning [21]. Another review of 12 randomized studies [20] concluded that the efficacy of computer–aided learning is reasonably well established. However, these authors also stressed that most of the included studies had methodological issues, eg, lack of power, attrition and a high risk of contamination. These methodological issues were still present in the studies we included in our review despite it being published a decade later.

There were also some differences between our results and the existing literature. Thirteen of the 14 included non–randomised controlled trials on the effect of computer–based instruction on knowledge and attitudes towards eLearning of health professions students favoured eLearning over traditional methods in another review [86]. Out of the 4 studies which compared students’ attitudes towards the intervention, 3 demonstrated that computer–based instruction students had more positive attitudes towards their instructional method than students exposed to conventional teaching [86]. Our findings were less positive towards offline eLearning and generally showed no difference in knowledge and attitudes between the intervention groups. This might potentially be explained by a larger presence of studies that did not blind the outcome assessment in the aforementioned review [86]. This could have resulted in students feeling more obliged to answer positively. In addition, the review assessed the subjective outcomes of attitudes and satisfaction, the assessment of which was very heterogeneous in the included studies [86], whereas we only assessed the results regarding students’ satisfaction and attitude that dealt with the difference between eLearning and traditional learning to keep the results as homogeneous as possible. Another systematic review [12] of 7 studies in allied health professions, medicine and nursing students reported that in all but 1 of the studies improvement in students’ competencies, clinical skills, self–efficacy and clinical reasoning was seen when blended learning was used. This review included a very heterogeneous sample of studies with both online and offline blended learning. It included both controlled trials and trials that were not. Also, this review excluded all studies that did not report methods or results sufficiently or properly [12]. Our review yielded a less positive conclusion, perhaps because we considered all studies regardless of quality to assess the full body of evidence. Furthermore, we had a more comprehensive search strategy allowing us to review a much larger number of studies. Because of these differences and the differences in topic, it is therefore not surprising that we reached different conclusions.

Our study has many strengths. First, we optimised the probability of identification of all relevant literature by conducting our search using sensitive search strategy, multiple recognised literature databases without imposing language restrictions as well as by screening references of the selected articles. To enhance data quality, every identified article was screened by 2 people independently, and their results were compared. The same applied to the data extraction of the selected articles, which was enhanced by using a standardised form for recording. The distinction between undergraduate and postgraduate education, and the focus on the former, increased the applicability of our results. The learning process at postgraduate level tends to be different, involving bedside learning and more in–depth exploration of the content. Additionally, patient outcomes are usually used as a proxy measure of the effectiveness of educational interventions in postgraduate education. An additional strength of our report was that our search resulted in the inclusion of both developed and developing countries. Finally, we followed the preferred reporting items for systematic reviews and meta–analyses (PRISMA), a framework tool used to set the minimum evidence–based items to be included when conducting and writing systematic reviews. Doing so and by using the Cochrane methodology maximized the completeness, transparency and accountability of our reporting of findings.

Despite its strengths, our systematic review also has some limitations. First, we were unable to identify unpublished studies. Second, we were unable to consider the pedagogical approach in more detail mainly because of the incomplete reporting of pedagogical methods within the included studies and because we did not request information on its details from the study authors. Third, our classification into offline eLearning and online eLearning and the other 3 categories is pragmatic and not an established classification. Other authors may suggest other groupings. However, eLearning remains a recent field in which the definitions, concepts, evaluation tools and measures still lack consensus [87]. Some of the studies categorised and analysed as offline eLearning were using eLearning interventions that were downloaded from WebCT Blackboard or sent to the students via email. This could be considered online eLearning, however, since the eLearning component could function fully offline and to avoid too much heterogeneity between the studies, we classified it as offline. Also, the mode of delivery of the eLearning material could have been replaced by an offline one (eg, CD ROM) and could therefore be used in areas with limited internet access.

Finally, our choice to include articles from 2000 onwards only could be challenged. However the choice of 2000 can be justified by a rise in the interest in eLearning illustrated in part by several national and international reports and publications on the topic from this year onwards. These more recent reports are likely to have used more modern forms of eLearning than older reports and are thus timelier, especially considering technological developments.

Furthermore, there were some limitations to the evidence that was available and included in this review. The lack of or insufficient reporting of results in some of the included studies resulted in the restricted level of detail in the analysis of certain outcomes of interest. Another important limitation to the evidence included is the lack of clarity of 1 or more aspects of the methodology used in the majority of the included trials and in some cases the occurrence of attrition. Although we contacted authors to obtain the missing information, some authors did not reply to our request and others did not know the answers. The lack of or insufficient reporting of methods and results lead to an inability to draw a robust conclusion allowing for generalisation to all undergraduate students around the world due to the study selection process and the limitations of the included studies.

We were unable to assess the cost–effectiveness of eLearning vs traditional learning because none of the identified studies formally assessed it. The 6 articles [27,34,35,53,68,70] that mentioned economic elements such as hours spent developing the program suggested that eLearning modules cost more to develop than using already established traditional learning methods, but also highlight that this can be done with limited resources.

None of the studies specifically addressed adverse effects of eLearning. This may be because potentially negative effects of eLearning that are regularly cited [24] focus on loneliness and depression, which could be regarded to be an aspect of students’ satisfaction and attitudes. Potential reduced efficacy and effectiveness of eLearning would have been evaluated as part of the assessment of skills and knowledge.

In summary, the findings from this systematic review suggest that offline eLearning is similar to traditional learning in terms of knowledge and skill acquisition and that it is possibly superior to traditional learning. In addition, they offer a more convenient, and more cost–effective, alternative to facilitate competency development and the training of health care professionals around the globe.

Our results indicate that students were more favourable towards the eLearning interventions. However, due to a high risk of bias these results should be interpreted with caution.

### Implications for policy makers

This systematic review indicates that offline eLearning is likely to be as effective as traditional learning, possibly superior and this presents a potential incentive for policy makers to encourage the development of offline eLearning curricula. These offline eLearning programs could potentially help address the health care worker shortage by contributing to greater access to education and training as part of scaling up the education of health workers especially in the developing world where internet access is limited and the need for an increase in the number of health professionals is greatest. However, there are still barriers (eg, computer access and access to eLearning material) that need to be overcome and this could be helped by changing policies and facilitating investments in ICT.

### Implications for educational institutions

Many eLearning programs were developed by local enthusiasts within universities and this review showed that these programs were likely to be effective in terms of knowledge and skills acquisition. Therefore, educational institutions should encourage such enthusiasts who wish to develop eLearning materials to improve the learning experience and knowledge and skills acquired by their students. Despite the fact that a robust conclusion on whether or not eLearning is superior to traditional learning could not be drawn we believe that educational institutions should not refrain from investing in offline eLearning material.

### Implications for future research

Offline eLearning is still likely to be a key player in education in the next decade where technology in education is expected to be used more and more and therefore researchers should continue to investigate the effects of this intervention on knowledge, skills and students’ satisfaction and attitudes especially in developing countries. Future individual studies should continue to improve the methodology (eg, avoid contamination and volunteer bias) with which the eLearning intervention is investigated and report their study according to the CONSORT guidelines.

Furthermore, we suggest that a well–defined and commonly used taxonomy for the different types and aspects of eLearning should be developed and employed in future research to enable easier comparison of different eLearning studies.

## References

[1] World Health Organization. Global health workforce shortage to reach 12.9 million in coming decades. 2013. Available from: http://www.who.int/mediacentre/news/releases/2013/health–workforce–shortage/en/# Accessed: 29 May 2014.

[2] World Health Organization. Working together for health: the World Health Report 2006. WHO Press. Geneva, 2006. Available from: http://whqlibdoc.who.int/publications/2006/9241563176_eng.pdf Accessed: 30 May 2014.

[3] The Global Workforce Alliance. Making Health Workers Count The Global Workforce Alliance 2012 Annual Health Report. Geneva: WHO, 2012.

[4] World Health Organization. Millennium Development Goals (MDGs). 2000. Available from: http://www.who.int/topics/millennium_development_goals/about/en/ Accessed: 28 May 2014.

[5] Ellis RA, Goodyear P. Students’ experiences of e–learning in higher education: the ecology of sustainable innovation. London: Taylor Francis, Routledge, 2010.

[6] Clarke T, Hermens A (2010). Corporate developments and strategic alliances in e–learning.. Educ Train.

[7] Herrington J, Reeves TC, Oliver R (2010). A guide to authentic e–learning.. Br J Educ Technol.

[8] Sangrà A, Vlachopoulos D, Cabrera N (2012). Building an inclusive definition of e–learning: An approach to the conceptual framework.. Int Rev Res Open Distance Learn.

[9] Masters K, Ellaway R (2008). e–Learning in medical education Guide 32 Part 2: Technology, management and design.. Med Teach.

[10] Duque G, Demontiero O, Whereat S, Gunawardene P, Leung O, Webster P (2013). Evaluation of a blended learning model in geriatric medicine: a successful learning experience for medical students.. Australas J Ageing.

[11] Zolfaghari M, Negarandeh R, Eybpoosh S (2013). Developing a blended learning program for nursing and midwifery students in Iran: Process and preliminary outcomes.. Iran J Nurs Midwifery Res..

[12] Rowe M, Frantz J, Bozalek V (2012). The role of blended learning in the clinical education of healthcare students: a systematic review.. Med Teach.

[13] Nartker AJ, Stevens L, Shumays A, Kalowela M, Kisimbo D, Potter K (2010). Increasing health worker capacity through distance learning: a comprehensive review of programmes in Tanzania.. Hum Resour Health.

[14] Makhdoom N, Khoshhal KI, Algaidi S, Heissam K, Zolaly MA (2013). “Blended learning” as an effective teaching and learning strategy in clinical medicine: a comparative cross–sectional university–based study.. J Taibah Univ Med Sci..

[15] Childs S, Blenkinsopp E, Hall A, Walton G (2005). Effective e–learning for health professionals and students––barriers and their solutions. A systematic review of the literature––findings from the HeXL project.. Health Info Libr J.

[16] Colace F, De Santo M, Pietrosanto A. Evaluation models for e–learning platform: an AHP approach. Frontiers in Education Conference, 36th Annual. IEEE; 2006. p. 1–6.

[17] International Telecommunication Union. ICT Facts & Figures 2013. The world in 2013 [Internet]. International Telecommunication Union Geneva. 2013 Available from: http://www.itu.int/en/ITU–D/Statistics/Pages/default.aspx. Accessed: 23 May 2014.

[18] Andersson A (2008). Seven major challenges for e–learning in developing countries: Case study eBIT, Sri Lanka Annika Andersson Örebro University, Sweden.. Int J Educ Dev Inf Commun Technol..

[19] Oye N, Salleh M, Iahad N (2011). Challenges of E–Learing in Nigerian University Education Based on the Experience of Developed Countries.. Int J Manag Inf Technol..

[20] Greenhalgh T (2001). Computer assisted learning in undergraduate medical education.. BMJ.

[21] Rosenberg H, Grad HA, Matear DW (2003). The effectiveness of computer–aided, self–instructional programs in dental education: a systematic review of the literature.. J Dent Educ.

[22] Higgins J, Green S, Collaboration C. Cochrane handbook for systematic reviews of interventions. 5.0.1 ed. London: Wiley–Blackwell, 2008.

[23] UNESCO Institute for Statistics. International Standard Classification of Education, ISCED 2011. Available from: https://www.stat.si/doc/sosvet/Sosvet_19/Sos19_s1925-2013.pdf. Accessed: 30 May 2014.

[24] Howell D (2001). Elements of effective e–learning: Three design methods to minimize side effects of online courses.. Coll Teach.

[25] Saadé RG, Kira D (2009). Computer anxiety in e–learning: the effect of computer self–efficacy development of research hypotheses.. J Inf Technol Educ.

[26] Rodgers M, Sowden A, Petticrew M, Arai L, Roberts H, Britten N (2009). Testing methodological guidance on the conduct of narrative synthesis in systematic reviews.. Evaluation.

[27] Ackermann O, Siemann H, Schwarting T, Ruchholtz S (2010). Z Orthop Unfall.

[28] Amesse LS, Callendar E, Pfaff–Amesse T, Duke J, Herbert WNP (2008). Evaluation of Computer–aided Strategies for Teaching Medical Students Prenatal Ultrasound Diagnostic Skills.. Med Educ Online.

[29] Armstrong P, Elliott T, Ronald J, Paterson B (2009). Comparison of traditional and interactive teaching methods in a UK emergency department.. Eur J Emerg Med.

[30] Bains M,, Reynolds PA, McDonald F, Sherriff M (2011). Effectiveness and acceptability of face–to–face, blended and e–learning: a randomised trial of orthodontic undergraduates.. Eur J Dent Educ.

[31] Bloomfield J, Roberts J, While A. (2010). The effect of computer–assisted learning versus conventional teaching methods on the acquisition and retention of handwashing theory and skills in pre–qualification nursing students: a randomised controlled trial.. Int J Nurs Stud.

[32] Boet S, Bould MD, Schaeffer R, Fischhof S, Stojeba N, Naik VN (2010). Learning fibreoptic intubation with a virtual computer program transfers to “hands on” improvement.. Eur J Anaesthesiol.

[33] Bogacki RE, Best A, Abbey LM (2004). Equivalence study of a dental anatomy computer–assisted learning program.. J Dent Educ.

[34] Bradley P, Oterholt C, Herrin J, Nordheim L, Bjřrndal A (2005). Comparison of directed and self–directed learning in evidence–based medicine: a randomised controlled trial.. Med Educ.

[35] Davis J, Crabb S, Rogers E, Zamora J, Khan K (2008). Computer–based teaching is as good as face to face lecture–based teaching of evidence based medicine: a randomized controlled trial.. Med Teach.

[36] Feeg VD, Bashatah A, Langley C (2005). Development and testing of a CD–ROM based tutorial for nursing students: getting ready for HIPAA.. J Nurs Educ.

[37] Gelb DJ (2001). Is newer necessarily better?: Assessment of a computer tutorial on neuroanatomical localization.. Neurology.

[38] Glicksman JT, Brandt MG, Moukarbel RV, Rotenberg B, Fung K (2009). Computer–assisted teaching of epistaxis management: a Randomized Controlled Trial.. Laryngoscope.

[39] Goldsworthy S, Lawrence N, Goodman W (2006). The use of personal digital assistants at the point of care in an undergraduate nursing program.. Comput Inform Nurs.

[40] Green MJ, Levi BH (2011). Teaching advance care planning to medical students with a computer–based decision aid.. J Cancer Educ.

[41] Holt RI, Miklaszewicz P, Cranston IC, Russell–Jones D, Rees PJ, Sönksen PH (2001). Computer assisted learning is an effective way of teaching endocrinology.. Clin Endocrinol (Oxf).

[42] Howerton WB, Platin E, Ludlow J, Tyndall DA (2002). The influence of computer–assisted instruction on acquiring early skills in intraoral radiography.. J Dent Educ.

[43] Hu A, Wilson T, Ladak H, Haase P, Doyle P, Fung K (2010). Evaluation of a three–dimensional educational computer model of the larynx: voicing a new direction.. J Otolaryngol Head Neck Surg..

[44] Hudson JN (2004). Computer–aided learning in the real world of medical education: does the quality of interaction with the computer affect student learning?. Med Educ.

[45] Jeffries PR, Woolf S, Linde B (2003). Technology–based vs. traditional instruction. A comparison of two methods for teaching the skill of performing a 12–lead ECG.. Nurs Educ Perspect.

[46] Jowett N, LeBlanc V, Xeroulis G, MacRae H, Dubrowski A (2007). Surgical skill acquisition with self–directed practice using computer–based video training.. Am J Surg.

[47] Kalet AL, Song HS, Sarpel U, Schwartz R, Brenner J, Ark TK (2012). Just enough, but not too much interactivity leads to better clinical skills performance after a computer assisted learning module.. Med Teach.

[48] Kim J, Chang S, Lee S, Jun E, Kim Y (2003). An experimental study of students’ self–learning of the San–Yin–Jiao pressure procedure using CD–ROM or printed materials.. J Nurs Educ.

[49] Kong J, Li X, Wang Y, Sun W, Zhang J (2009). Effect of digital problem–based learning cases on student learning outcomes in ophthalmology courses.. Arch Ophthalmol.

[50] Kurihara Y, Kuramoto S, Matsuura K, Miki Y, Oda K, Seo H (2004). Academic performance and comparative effectiveness of computer– and textbook–based self–instruction.. Stud Health Technol Inform.

[51] Lira RPC, Felix JPF, Chaves FRP, Fulco EAM, de Carvalho KMM, Zimmermann A (2013). E–learning as a complement to presential teaching of blindness prevention: a randomized clinical trial. Rev Bras Oftalmol..

[52] Maleck M, Fischer MR, Kammer B, Zeiler C, Mangel E, Schenk F (2001). Do computers teach better? A media comparison study for case–based teaching in radiology.. Radiographics.

[53] McDonough M, Marks IM (2002). Teaching medical students exposure therapy for phobia/panic – randomized, controlled comparison of face–to–face tutorial in small groups vs. solo computer instruction.. Med Educ.

[54] McMullan M, Jones R, Lea S (2011). The effect of an interactive e–drug calculations package on nursing students’ drug calculation ability and self–efficacy. Int J Med Inform..

[55] Miedzybrodzka Z, Hamilton NM, Gregory H, Milner B, Frade I, Sinclair T (2001). Teaching undergraduates about familial breast cancer: comparison of a computer assisted learning (CAL) package with a traditional tutorial approach.. Eur J Hum Genet.

[56] Morgulis Y, Kumar RK, Lindeman R, Velan GM (2012). Impact on learning of an e–learning module on leukaemia: a randomised controlled trial.. BMC Med Educ.

[57] Nance ET, Lanning SK, Gunsolley JC (2009). Dental anatomy carving computer–assisted instruction program: an assessment of student performance and perceptions.. J Dent Educ.

[58] Nola M, Morović A, Dotlić S, Dominis M, Jukić S, Damjanov I (2005). Croatian implementation of a computer–based teaching program from the University of Kansas, USA.. Croat Med J.

[59] Nousiainen M, Brydges R, Backstein D, Dubrowski A (2008). Comparison of expert instruction and computer–based video training in teaching fundamental surgical skills to medical students.. Surgery.

[60] Perfeito JAJ, Forte V, Giudici R, Succi JE, Lee JM, Sigulem D (2008). Desenvolvimento e avaliaçăo de um programa multimídia de computador para ensino de drenagem pleural. J Bras Pneumol.

[61] Prinz A, Bolz M, Findl O (2005). Advantage of three dimensional animated teaching over traditional surgical videos for teaching ophthalmic surgery: a randomised study.. Br J Ophthalmol.

[62] Pusic MV, Leblanc VR, Miller SZ (2007). Linear versus web–style layout of computer tutorials for medical student learning of radiograph interpretation.. Acad Radiol.

[63] Qayumi AK, Kurihara Y, Imai M, Pachev G, Seo H, Hoshino Y (2004). Comparison of computer–assisted instruction (CAI) versus traditional textbook methods for training in abdominal examination (Japanese experience).. Med Educ.

[64] Roppolo LP, Heymann R, Pepe P, Wagner J, Commons B, Miller R (2011). A randomized controlled trial comparing traditional training in cardiopulmonary resuscitation (CPR) to self–directed CPR learning in first year medical students: The two–person CPR study.. Resuscitation.

[65] Seabra D, Srougi M, Baptista R, Nesrallah LJ, Ortiz V, Sigulem D (2004). Computer aided learning versus standard lecture for undergraduate education in urology.. J Urol.

[66] Shomaker TS, Ricks DJ, Hale DC (2002). A prospective, randomized controlled study of computer–assisted learning in parasitology.. Acad Med.

[67] Solomon DJ, Ferenchick GS, Laird–Fick HS, Kavanaugh K (2004). A randomized trial comparing digital and live lecture formats [ISRCTN40455708.. BMC Med Educ.

[68] Tunuguntla R, Rodriguez O, Ruiz JG, Qadri SS, Mintzer MJ, Van Zuilen MH (2008). Computer–based animations and static graphics as medical student aids in learning home safety assessment: a randomized controlled trial.. Med Teach.

[69] Vichitvejpaisal P, Sitthikongsak S, Preechakoon B, Kraiprasit K, Parakkamodom S, Manon C (2001). Does computer–assisted instruction really help to improve the learning process?. Med Educ.

[70] Vivekananda–Schmidt P, Lewis M, Hassell AB (2005). Cluster randomized controlled trial of the impact of a computer–assisted learning package on the learning of musculoskeletal examination skills by undergraduate medical students.. Arthritis Rheum.

[71] Weih M, Triebner S, Heckmann J, Segarra L, Hahn E, Kornhuber J (2008). Fortschr Neurol Psychiatr.

[72] Williams C, Aubin S, Harkin P, Cottrell D (2001). A randomized, controlled, single–blind trial of teaching provided by a computer–based multimedia package versus lecture.. Med Educ.

[73] Xeroulis GJ, Park J, Moulton C-A, Reznick RK, Leblanc V, Dubrowski A (2007). Teaching suturing and knot–tying skills to medical students: a randomized controlled study comparing computer–based video instruction and (concurrent and summary) expert feedback.. Surgery.

[74] Fritz D, Hu A, Wilson T, Ladak H, Haase P, Fung K (2011). Long–term retention of a 3–dimensional educational computer model of the larynx: a follow–up study.. Arch Otolaryngol Head Neck Surg.

[75] Johnston R, Hepworth J, Goldsmith M, Lacasse C (2010). Use of iPod^TM^ technology in medical–surgical nursing courses: effect on grades.. Int J Nurs Educ Scholarsh.

[76] Eng J (2012). Teaching receiver operating characteristic analysis: an interactive laboratory exercise.. Acad Radiol.

[77] Fincher RE, Abdulla A (1988). Computer–assisted learning compared with weekly seminars for teaching fundamental electrocardiography to junior medical students.. South Med J.

[78] Garrett TJ, Ashford A, Savage D (1987). A comparison of computer–assisted instruction and tutorials in hematology and oncology.. J Med Educ.

[79] Rouse DP (2000). The effectiveness of computer–assisted instruction in teaching nursing students about congenital heart disease.. Comput Nurs.

[80] Tews M, Brennan K, Begaz T, Treat R (2011). Medical student case presentation performance and perception when using mobile learning technology in the emergency department.. Med Educ Online.

[81] Willett G. A comparative evaluation of teaching methods in an introductory neuroscience course for physical therapy students. University of Nebraska Medical Center; 2006. p. 125. 19753397

[82] Johnston JM, Leung G, Tin K (2004). Evaluation of a handheld clinical decision support tool for evidence–based learning and practice in medical undergraduates.. Med Educ.

[83] Guy JF, Frisby A (1992). Using interactive videodiscs to teach gross anatomy to undergraduates at the Ohio State University.. Acad Med.

[84] Pusic MV, Pachev GS, MacDonald WA (2007). Embedding medical student computer tutorials into a busy emergency department.. Acad Emerg Med.

[85] Devitt P, Palmer E (1999). Computer–aided learning: an overvalued educational resource?. Med Educ.

[86] Cohen P, Dacanay L (1992). Computer–based instruction and health professions education: A meta–analysis of outcomes.. Eval Health Prof.

[87] Chua BB, Dyson LE. Applying the ISO9126 model to the evaluation of an e–learning system. Beyond the comfort zone: proceedings of the 21st ascilite conference. Perth; 2004. p. 184–90.

