# Supplementary material for: Offline eLearning for undergraduates in health professions: A systematic review of the impact on knowledge, skills, attitudes and satisfaction
Source: J Glob Health. 2014 Jun;4(1):010405. doi: 10.7189/jogh.04.010405 (PMC4073241; doi:10.7189/jogh.04.010405)
Supplement: Online Supplementary Document [file jogh-04-010405-s001.pdf]

Online Supplementary Document

Rasmussen et al. Offline eLearning for undergraduates in health professions: A systematic review of the impact on knowledge, skills, attitudes and satisfaction
J Glob Health 2014;4:010405

1: Definition of the six eLearning categories

Six broad categories of eLearning interventions, based on the technologies employed. The categories were defined as follows:

- Offline computer-based eLearning: standalone applications where internet or intranet connections are not required for the delivery of the learning activities. The main tasks of the eLearning software in this category are usually performed on a PC or laptop. The delivery channels are usually CD-ROM or USB memory sticks. Alternatively, the delivery mode of the software can be via a networked connection, as long as the learning activities do not rely on this connection.
- Online and local area network-based eLearning: interventions that use the transmission control protocol (TCP) and the internet protocol (IP) to provide the full functionalities of the educational intervention. As implied by the terminology used, the delivery channels are usually the internet or a local area network.
- Psychomotor skills trainer: technology that will develop fine motor coordination skills and techniques in education, such as the precise use of instruments of tools.
- Virtual reality environments: computer-generated representations of a real or artificial environment. This can be interacted with by external involvement, allowing for a first-person active learning experience.
- Digital game-based learning: the application of game principles and mechanics in non-game contexts to engage users in solving problems and improve their engagement, attitudes, motivation and knowledge.
- mLearning: any eLearning intervention that uses handheld, mobile devices to deliver educational content such as a mobile phone, iPod or tablet.

2: Fields included in the data extraction form

1. Study ID

2.1. Journal where the study was published

2.2. Type of publication

2.3. Authors' affiliation

3.1. Study design as specified in the report

3.2. Study aims & objectives

3.3. Countries where the study was conducted

3.4. WHO region

3.5.World Bank income category

3.6. Study start date

3.7. Study end date

3.8. Method of comparison

4.1. Total number of participants invited to take part in the study

4.2. Total number of participants who agreed to take part in the study

4.3. Total number of participants meeting the inclusion criteria for participation in the study

4.4. Total number of participants included in the study

4.5. If cluster RCT, total number of clusters initially included in the study

4.6. If cluster RCT, total number of clusters randomised

4.7. Inclusion criteria

4.8. Exclusion criteria

5.1. Total number of experimental groups (including the control group)

5.2. Were groups tested for baseline differences?

5.2.1. If there were baseline differences, please specify what the difference was

5.3. Indicate the type of degree or qualification that participants were pursuing

If other, please specify:

5.4. Year of study within the anticipated degree or qualification

5.5. Control group

5.5.1. Total number of participants/clusters allocated to the control group

5.5.2. Mean age (standard deviation) of the participants in the control group

5.5.3. Name of educational intervention used as control

5.5.4. Description of the control condition

5.5.5. Field of study

5.6.6. Exposure to the control condition during the whole study

5.5.7. Total exposure time to the intervention

5.5.8. Type of technology/devices used to deliver the intervention

5.5.9. Delivery approach of the intervention

If other, please specify:

5.5.10. Was the usual delivery mode of the assessment changed?

5.5.11. If yes, please specify

5.5.12. Was the delivery mode of the assessment uniform across all the experimental groups?

5.6. Intervention group I

5.6.1. Total number of participants/clusters allocated to this intervention group.

5.6.2. Mean age (standard deviation) of the participants in this intervention group

5.6.3. Name of educational intervention used in this intervention group

5.6.4. Description of this intervention condition

5.6.5. Field of study

5.6.6. Exposure to this intervention condition during the whole study

5.6.7. Total exposure time to the intervention

5.6.8. Type of technology/devices used to deliver the intervention

5.6.9. Delivery approach of the intervention

If other, please specify:

5.6.10. Was the usual delivery mode of the assessment changed?

5.6.11. If yes, please specify

5.6.12. Was the delivery mode of the assessment uniform across all the experimental groups?

5.7. Intervention group II

5.7.1. Total number of participants/clusters allocated to this intervention group.

5.7.2. Mean age (standard deviation) of the participants in this intervention group

5.7.3. Name of educational intervention used in this intervention group

5.7.4. Description of this intervention condition

5.7.5. Field of study

5.7.6. Exposure to this intervention condition during the whole study

5.7.7. Total exposure time to the intervention

5.7.8. Type of technology/devices used to deliver the intervention

5.7.9. Delivery approach of the intervention

If other, please specify:

5.7.10. Was the usual delivery mode of the assessment changed?

5.7.11. If yes, please specify

5.7.12. Was the delivery mode of the assessment uniform across all the experimental groups?

5.8. Intervention group III

5.8.1. Total number of participants/clusters allocated to this intervention group.

5.8.2. Mean age (standard deviation) of the participants in this intervention group

5.8.3. Name of educational intervention used in this intervention group

5.8.4. Description of this intervention condition

5.8.5. Field of study

5.8.6. Exposure to this intervention condition during the whole study

5.8.7. Total exposure time to the intervention

5.8.8. Type of technology/devices used to deliver the intervention

5.8.9. Delivery approach of the intervention

If other, please specify:

5.8.10. Was the usual delivery mode of the assessment changed?

5.8.11. If yes, please specify

5.8.12. Was the delivery mode of the assessment uniform across all the experimental groups?

If more than 4 intervention groups (including the control group), please copy and paste the relevant cells as needed

6.1. Was 'Knowledge' measured? - If not, please go to section 6.2.

6.1.1. Instrument or measure used to asses knowledge - as specified by the study authors

6.1.2. Is this a validated instrument?

6.2. Were 'Skills' measured? - If not, please go to section 6.3.

6.2.1. Instrument or measure used to asses skills - as specified by the study authors

6.2.2. Is this a validated instrument?

6.3. Were 'Attitudes' measured? - If not, please go to section 6.4.

6.3.1. Instrument or measure used to asses attitudes - as specified by the study authors

6.3.2. Is this a validated instrument?

6.4. Was 'Student satisfaction' measured? - If not, please go to section 6.5.

6.4.1. Instrument or measure used to asses student satisfaction - as specified by the study authors

6.4.2. Is this a validated instrument?

6.5. Was an economic evaluation of the eLearning intervention performed?

6.5.1. Were quantitative indicators like costs, investments, hardware, software, license fees and benefits/savings of the eLearning intervention measured?

6.5.2. Was the urgency of the eLearning intervention (i.e., due to a new regulation or organisational demand) mentioned?

6.5.3. Were qualitative-strategic indicators of the eLearning intervention like quality and performance improvements measured?

6.5.4. Were external factors of the eLearning intervention like synergy effects or economies of scope measured?

6.5.5. Please list any additional economic indicators that were measured

7.1. Selection bias

7.1.1. Random sequence generation

7.1.1.1. Describe the method used to generate the allocation sequence in sufficient detail to allow an assessment of whether it should produce comparable groups

7.1.1.2. Please indicate your judgement

7.1.2. Allocation concealment

7.1.2.1. Describe the method used to conceal the allocation sequence in sufficient detail to determine whether intervention allocations could have been foreseen in advance of, or during, enrolment

7.1.2.2. Please indicate your judgement

7.2. Performance bias

7.2.1. Blinding of participants and personnel

7.2.1.1. Describe all measures used, if any, to blind study participants and personnel from knowledge of which intervention a participant received. Provide any information relating to whether the intended blinding was effective

7.2.1.2. Please indicate your judgement

7.3. Detection bias

7.3.1. Blinding of outcome assessment

7.3.1.1. Describe all measures used, if any, to blind outcome assessors from knowledge of which intervention a participant received. Provide any information relating to whether the intended blinding was effective

7.3.1.2. Please indicate your judgement

7.4. Attrition bias

7.4.1. Incomplete outcome data

7.4.1.1. Describe the completeness of outcome data for each main outcome, including attrition and exclusions from the analysis. State whether attrition and exclusions were reported, the numbers in each intervention group (compared with total randomized participants), reasons for attrition/exclusions where reported, and any re-inclusions in analyses performed by the review authors

7.4.1.2. Please indicate your judgement

7.5. Reporting bias

7.5.1. Selective reporting

7.5.1.1. State how the possibility of selective outcome reporting was examined by the review authors, and what was found.

7.5.1.2. Please indicate your judgement

7.6. Other bias

7.6.1. Other source of bias

7.6.1.1. State any important concerns about bias not addressed in the other domains in the tool

7.6.1.2. Please indicate your judgement

8.1. Recruitment bias

8.1.1. Please describe any evidence of recruitment bias.

8.2. Baseline imbalances

8.2.1. Please describe any evidence of baseline imbalances.

8.3. Loss of clusters

8.3.1. Please indicate any evidence of risk of bias due to loss of clusters.

8.4. Incorrect analysis

8.4.1. Please indicate any evidence of incorrect analysis.

9.1. Control group

9.1.1. Outcome reported

9.1.2. Measure of effect size (as measured by the study authors)

9.1.3. Measure of dispersion (as measured by the study authors)

9.1.1. Outcome reported

9.1.2. Measure of effect size (as measured by the study authors)

9.1.3. Measure of dispersion (as measured by the study authors)

9.1.1. Outcome reported

9.1.2. Measure of effect size (as measured by the study authors)

9.1.3. Measure of dispersion (as measured by the study authors)

9.1.1. Outcome reported

9.1.2. Measure of effect size (as measured by the study authors)

9.1.3. Measure of dispersion (as measured by the study authors)

If more than one outcome was reported, please insert more cells here and copy and paste the relevant data entry boxes.

9.2. Intervention I group

9.2.1. Outcome reported

9.2.2. Measure of effect size (as measured by the study authors)

9.2.3. Measure of dispersion (as measured by the study authors)

9.2.1. Outcome reported

9.2.2. Measure of effect size (as measured by the study authors)

9.2.3. Measure of dispersion (as measured by the study authors)

9.2.1. Outcome reported

9.2.2. Measure of effect size (as measured by the study authors)

9.2.3. Measure of dispersion (as measured by the study authors)

9.2.1. Outcome reported

9.2.2. Measure of effect size (as measured by the study authors)

9.2.3. Measure of dispersion (as measured by the study authors)

If more than one outcome was reported, please insert more cells here and copy and paste the relevant data entry boxes.

9.3. Intervention II group

9.3.1. Outcome reported

9.3.2. Measure of effect size (as measured by the study authors)

9.3.3. Measure of dispersion (as measured by the study authors)

9.3.1. Outcome reported

9.3.2. Measure of effect size (as measured by the study authors)

9.3.3. Measure of dispersion (as measured by the study authors)

9.3.1. Outcome reported

9.3.2. Measure of effect size (as measured by the study authors)

9.3.3. Measure of dispersion (as measured by the study authors)

9.3.1. Outcome reported

9.3.2. Measure of effect size (as measured by the study authors)

9.3.3. Measure of dispersion (as measured by the study authors)

If more than one outcome was reported, please insert more cells here and copy and paste the relevant data entry boxes.

9.4. Intervention III group

9.4.1. Outcome reported

9.4.2. Measure of effect size (as measured by the study authors)

9.4.3. Measure of dispersion (as measured by the study authors)

9.4.1. Outcome reported

9.4.2. Measure of effect size (as measured by the study authors)

9.4.3. Measure of dispersion (as measured by the study authors)

9.4.1. Outcome reported

9.4.2. Measure of effect size (as measured by the study authors)

9.4.3. Measure of dispersion (as measured by the study authors)

If more than one outcome was reported, please insert more cells here and copy and paste the relevant data entry boxes.

9.5. Comparison I

9.5.1. Please indicate the intervention groups being compared

9.5.2. Please indicate the outcomes being compared

9.5.3. Statistical test used for the comparison

9.5.4. Result of the test

9.5.5. P value / Confidence intervals

9.6. Comparison II

9.6.1. Please indicate the intervention groups being compared

9.6.2. Please indicate the outcomes being compared

9.6.3. Statistical test used for the comparison

9.6.4. Result of the test

9.6.5. P value / Confidence intervals

9.7. Comparison III

9.7.1. Please indicate the intervention groups being compared

9.7.2. Please indicate the outcomes being compared

9.7.3. Statistical test used for the comparison

9.7.4. Result of the test

9.7.5. P value / Confidence intervals

9.8. Comparison IV

9.8.1. Please indicate the intervention groups being compared

9.8.2. Please indicate the outcomes being compared

9.8.3. Statistical test used for the comparison

9.8.4. Result of the test

9.8.5. P value / Confidence intervals

9.9. Comparison V

9.9.1. Please indicate the intervention groups being compared

9.9.2. Please indicate the outcomes being compared

9.9.3. Statistical test used for the comparison

9.9.4. Result of the test

9.9.5. P value / Confidence intervals

9.9. Comparison V

9.9.1. Please indicate the intervention groups being compared

9.9.2. Please indicate the outcomes being compared

9.9.3. Statistical test used for the comparison

9.9.4. Result of the test

9.9.5. P value / Confidence intervals

9.9. Comparison V

9.9.1. Please indicate the intervention groups being compared

9.9.2. Please indicate the outcomes being compared

9.9.3. Statistical test used for the comparison

9.9.4. Result of the test

9.9.5. P value / Confidence intervals

For each comparison conducted in the study, please copy and paste the cells as appropriate

10.1. Organisational setting

10.2. Technological infrastructure

10.3. Instructional Systems Design and Curriculum development

10.4. Delivery

10.5. Advantages of eLearning - as reported by the study authors

10.6. Disadvantages of eLearning - as reported by the study authors

11.1. Source of financing - as reported by the study authors

11.2. Did the intervention undergo a formal accreditation process within the host institution?

11.3. If yes, please describe

11.4. Was the eLearning intervention developed for this study consequently adopted as a formal method for the delivery of education at the host institution?

11.5. If yes, please specify

12.1. Study conclusions - as stated by the study authors

12.2. Limitations of the study - as reported by the study authors

12.3. Was contact with the study authors sought? - If No, please go to section 12.5

12.4. Please indicate the nature of the information requested from the study authors

12.5. Please indicate the results of the request for information

12.6. Additional notes

3: Results of electronic searches

Number of citations yielded by the electronic searches for each bibliographic database

Database	Results	
	Before de-duplication	After de-duplication
**MEDLINE**	941	806
**EMBASE**	3206	3123
**PsycINFO**	334	334
**Web of Knowledge**	6993	4099
**ERIC**	146	146
**CENTRAL**	588	584
**Total**	**12208**	**9092**

4: Characteristics of included studies for offline computer-based eLearning

	Methods			Participants			Interventions		
STUDY ID	STUDY DESIGN	LOCATION	COMPARISON	TOTAL NUMBER	YEAR OF STUDY	HEALTHCARE SPECIALITY	CHARACTERISTICS	TIME AND TECHNOLOGY	TEST / OUTCOMES
Ackermann 2010	RCT	Germany	eLearning vs. traditional learning	19	Third year	Medicine	CG: students were given conventional learning material, access to the library and internet<br>IG: students were given software CD for installation on home PC	• 1 week (for intervention group 6 hours within 1 week)<br>• CD-ROM	Knowledge: MCQ, X-ray interpretation skills
Arnesse 2008	RCT	USA	eLearning vs. traditional learning	36	Third year	Medicine	CG: students were given paper-based tutorial session<br>IG: students were given computer-based learning tutorial session, composed of real-time video segments as well as audio and interactive components	• 90 minutes<br>• IBM ThinkPad laptop, CD-ROM	Knowledge: 34 fill-in-the-blank style questions, 2 equivalent examinations (test 1 consisted of 22 computer-posed questions and 12 paper-written questions. Test 2 consisted of 12 computer-posed questions and 22 paper-written questions)
Armstrong 2009	RCT	UK	eLearning vs. traditional learning	21	Fourth year	Medicine	CG: students were given lecture version of the eLearning tutorial, presented in a didactic form<br>IG: students were given interactive slide show of a blood gas interpretation tutorial	• time N/A (during 1 week, probably only 1 lecture/tutorial)<br>• Microsoft® PowerPoint®	Knowledge: 5 MCQ
Bains 2011	cRCT	UK	eLearning vs. traditional learning	90	Fourth year	Dentistry	CG: students were given teacher-led tutorial (face-to face-learning)<br>IG I: students were given online tutorial with no teacher (animated learner-controlled didactic program)<br>IG II: students were given online tutorial with no teacher followed by teacher-led tutorial<br>IG III: students were given teacher-led tutorial followed by online tutorial with no teacher	• 45 minutes (2 times 45 min for intervention groups II and III)<br>• WebCT® version 3.8	Knowledge: 10 MCQ<br>Attitudes: Likert scales and focus groups
Bloomfield 2010	RCT	UK	eLearning vs. traditional learning	223	First year	Nursing	CG: students were given conventional learning with a standardised teaching pack: a set of lecture notes; a set of black and white overhead transparency slides; the handwashing demonstration video; and a list of additional reference material. Following a short lecturer-led presentation, participants watched the video and were then offered the opportunity to practice the recommended handwashing technique<br>IG: students worked independently through a self-directed CAL module via an individual computer terminal in an on-campus computer room. The theoretical content was identical to that of the conventional teaching session and interactive activities; animated multimedia, high-quality photographs and links to relevant websites were also included to stimulate interest and promote learner engagement. The handwashing demonstration video was embedded within the module	• 90 minutes<br>• Computer, CAL module	Knowledge: 20 MCQ<br>Skills: OSCE
Boet 2010	N/A	France	eLearning vs. traditional learning	42	N/A	Medicine	CG: students were given conventional institutional didaction instruction lecture<br>IG: students were given conventional institutional didactic instruction lecture plus virtual fibreoptic intubation CD-ROM (developed from reconstructed images recreating the 3D environment of the airway)	• 1 hour lecture time (CD-ROM exposure was within 2 weeks, no measure of exposure time)<br>• A virtual multimedia simulator, the 'virtual fibreoptic intubation,' computer	Skills: ability to perform an intubation, primary endpoint being success within 4 minutes, evaluations were done in real time by the investigator

STUDY ID	Methods			Participants			Interventions		
	STUDY DESIGN	LOCATION	COMPARISON	TOTAL NUMBER	YEAR OF STUDY	HEALTHCARE SPECIALITY	CHARACTERISTICS	TIME AND TECHNOLOGY	TEST / OUTCOMES
Bogacki 2004	RCT	USA	eLearning vs. traditional learning	45	First year	Dentistry	CG: students were given traditional dental anatomy lecture<br>IG: students were given tooth morphology program (text, photographic images, illustrations, and lectures to teach morphology of the adult dentition)	• 6 week course<br>• CD-ROM and computer	Knowledge: exam
Bradley 2005	RCT	Norway	eLearning vs. traditional learning	168	Tenth semester	Medicine	CG: students were given 5 half-day workshops<br>IG: training relied on CAL, mainly using an English-language CD-ROM (and accompanying workbook). The CD-ROM consisted of 5 modules; course notes and interactive exercises that posed questions and gave automatic feedback on answers, checklists to appraise articles, a glossary of terms, several sample articles to appraise, and links to key internet sites. The accompanying workbook included all the necessary source material including several additional examples of scientific articles to appraise, further exercises, references, and checklists to appraise them. Because the workbook was in English, it was supplemented with non-interactive internet pages in Norwegian. The internet resource contained a glossary of terms, checklists to appraise articles, and further references. In addition, tutors (1 clinical epidemiologist and 1 librarian) were available at 5 specified teaching sessions lasting 3 hours	• 5 half days<br>• CD-ROM, PC, access to internet site	Knowledge: 7 MCQ and critical appraisal of a scientific paper<br>Attitudes: Likert scale
Davis 2008	RCT	UK	eLearning vs. traditional learning	179	First year	Medicine	CG: students were given standard lecture<br>IG: students were given computer-based learning (recording of the lecture, plus PowerPoint® presentation, plus internet links)	• 40 minutes<br>• PC, headphones, CD-ROM	Knowledge: 5 questions (2 structured and 3 MCQ)
Feeg 2005	RCT	USA	eLearning vs. traditional learning	91	N/A	Nursing	CG: students were given journal article<br>IG: students were given CD-ROM based tutorial plus journal article	• 2 weeks (within this intervention group spent on average 28.7 minutes<br>• PC, headphones, CD-ROM	Knowledge: 20 MCQ (18 used for analysis)
Gelb 2001	RCT	USA	eLearning vs. traditional learning	107	N/A	Medicine	CG: students were given a print version of the tutorial, containing the same information as computer tutorial<br>IG: students were given computer tutorial	• 8 months<br>• computer	Knowledge: MCQ
Glicksman 2009	RCT	Canada	eLearning vs. traditional learning	47	First year	Medicine	CG: students were given instructions and a quiet location for reading the article<br>IG: students were given CAL module designed wih identical content to the article	• time N/A<br>• computer, a program was designed using Articulate Presenter® a program that turns PowerPoint® presentations into Adobe® Flash®-based computer and web modules that run in a web browser	**Skills:** the time taken to pack the nose was measured in a standardised manner; videotape analysis using a previously validated global rating system adapted for the present study (7 outcomes, including respect for tissue, time and motion, instrument handling, flow of operation, knowledge of procedure, overall performance, and quality of final product, each based on a 5-point Likert scale); a checklist modeled on a previously validated human reliability assessment tool was used (6 items for the tampon pack and 8 items for the formal pack)<br>**Attitude:** questionnaire

STUDY ID	Methods			Participants			Interventions		
	STUDY DESIGN	LOCATION	COMPARISON	TOTAL NUMBER	YEAR OF STUDY	HEALTHCARE SPECIALITY	CHARACTERISTICS	TIME AND TECHNOLOGY	TEST / OUTCOMES
Weih 2008	RCT	Germany	eLearning vs. traditional learning	101	Eighth semester	Medicine, psychology	**CG:** traditional lecture<br>**IG:** CD-ROM, interactive LMS (LearnCube) in addition to traditional lecture	• traditional lecture 2 times 45 minutes, 1.3 hours for the programme<br>• home PC, Pentium II MS Windows 98 SE, LearnCube-Software	**Knowledge:** 20 MCQ<br>**Satisfaction:** 20 item questionnaire
Williams 2001	RCT	UK	eLearning vs. traditional learning	163	Fourth year	Medicine	**CG:** lecture with videotape, covering a detailed history of the presenting complaint and a mental state examination<br>**IG:** excerpts from the same video were used in the computer-based package. Video clips within the package show the referrer assessing the presenting problem and the patient describing their own symptoms, and provide key background information. Students are prompted to seek further information by carrying out physical, social and psychological investigations, and further video clips allow the learner to carry out a structured mental state examination before making a differential diagnosis. Users of the program can go at their own pace (although in this study, time was limited to match that available for students receiving the lecture) and navigate their own way through the package. Learners get regular feedback on the decisions they are making and are able to test their knowledge and skills in recognising mental state phenomena	• 55 minutes<br>• computer based package, computer	**Knowledge:** 10 5-stem MCQ and mental state exam
Xeroulis 2007	RCT	Canada	eLearning vs. traditional learning	60	First year	Medicine	**CG:** no additional intervention (control)<br>**IG I:** self-study with computer based video instructions, students could interact with the program in continuous replay and slow motion replay modules, watch the skills in their entirety, or access only sections specific to their learning needs<br>**IG II:** expert feedback during practice trials (concurrent feedback)<br>**IG III:** expert feedback after practice trials (summary feedback)	• 1 hour<br>• video instruction tool	**Skills:** global rating scale and Imperial College Surgical Assessment Device

CG = Control group
IG = Intervention group

*Publication contained two studies

STUDY ID	Methods			Participants			Interventions		
	STUDY DESIGN	LOCATION	COMPARISON	TOTAL NUMBER	YEAR OF STUDY	HEALTHCARE SPECIALITY	CHARACTERISTICS	TIME AND TECHNOLOGY	TEST / OUTCOMES
Jeffries 2003	RCT	USA	eLearning vs. traditional olearning	73	N/A	Nursing	**CG:** students were given self study module, brief lecture, demonstration by an instructor and hands-on experience<br>**IG:** students were given interactive CD-ROM in the laboratory computer cluster, embeded with virtual reality and supplemented with a self-study module	• 90 minutes for control group, 2 days (at times scheduled) for intervention group<br>• interactive CD-ROM in the laboratory computer cluster, embeded with virtual reality and supplemented with a self-study module	**Knowledge:** 27 MCQ<br>**Skills:** weighted, 22-item skills competency checklist<br>**Attitude:** Likert scale<br>**Satisfaction:** questionnaire
Jowett 2007	RCT	Canada	eLearning vs. eLearning	30	N/A	Medicine	**CG:** no intervention<br>**IG:** students were able to attend additional practice; 4 extra 3-minute practice blocks separated by video-captured trials with hand-motion tracking	• 0 and 12 minutes<br>• laptop, hand-motion tracking device	**Skills:** expert global rating scale; time; number of hand movements; path length
Kalet2012	RCT	USA	eLearning vs. eLearning	143	Second year	Medicine	**CG:** students were given online module, watch condition (students controlled only the pace of the presentation)<br>**IG I:** students were given online module, click condition (students used the mouse to trigger animated demonstrations)<br>**IG: II:** students were given online module, drag condition (students were able to click and drag tools in motions simulating actual performance of the task)	• 20 minutes<br>• computer-based multimedia presentation	**Knowledge:** 17 or 18 item MCQ<br>**Skills:** standardised patient checklist, patient note
Kim 2003	RCT	South Korea	eLearning vs. traditional olearning	75	Third year	Nursing	**CG:** students were given printed material, self learning<br>**IG:** students were given computer software, self learning	• 1 week, control group 55 +/-30 hours and intervention group 48.5 +/-27.8 hours<br>• CD-ROM, computer	**Knowledge:** assesment of theoretical background and concrete methods of applying pressure<br>**Skills:** checklist based on the steps of the procedure, student psychomotor skills and compentency when students applied pressure<br>**Attitude:** questionnaire<br>**Satisfaction:** questionnaire
Kong 2009	RCT	China	eLearning vs. traditional olearning	90	Fifth year	Medicine	**CG:** students were given conventional teaching, didactic model<br>**IG I:** students were given PBL teaching with paper based case description,<br>**IG II:** students were given PBL teaching with digital format material	• 2 hours twice weekly (total 18 hours)<br>• educational websites, and multimedia CD-ROM	**Knowledge:** theoretical and case analysis examinations<br>**Skills:** evaluation of students' practice<br>**Satisfaction:** questionnaire
Kurihara 2004	RCT	Japan	eLearning vs. traditional olearning	59	Third year	Medicine	**CG:** students were given traditional textbook learning<br>**IG I:** students were assigned to computer-based learning with CAI software cyberPatient<br>**IG II:** students were assigned to traditional text-book learning combined with cyberPatient<br>**IG III:** no intervention	• 4 hours<br>• cyberPatient: multimetida software. Multimedia software that consists of patient simulation models and special clinical skills learning modules. The abdominal physical examination learning module was used for this intervention	**Knowledge:** 40 MCQ (8 exluded for the analysis)<br>**Skills:** OSCE
Lira 2013	RCT	Brazil	eLearning vs. traditional olearning	68	Fourth year	Medicine	**CG:** students were assigned to the lecture<br>**IG** students were assigned to the lecture plus additional PDF article, sent a week before the class	• time N/A<br>• computer, internet, PDF	**Knowledge:** 7 MCQ

STUDY ID	Methods			Participants			Interventions		
	STUDY DESIGN	LOCATION	COMPARISON	TOTAL NUMBER	YEAR OF STUDY	HEALTHCARE SPECIALITY	CHARACTERISTICS	TIME AND TECHNOLOGY	TEST / OUTCOMES
Maleck 2001	RCT	Germany	eLearning vs. traditional learning	192	Third year	Medicine	**CG:** students were given paper version of the cases with the original film radiographs, option to attend the lecture and using textbook<br>**IG I:** students were assigned to computer-based cases along with interactive elements (MCQ, free text questions, drag-and-drop mapping tool), option to attend the lecture and use textbook<br>**IG II:** computer-based cases but without the interactive elements, option to attend the lecture and use textbook<br>**IG III:** no intervention, option to attend the lecture and use textbook	• 2 hours, the computer-based cases took 20 to 30 minutes per case<br>• Macintosh Power PC 8200/120 (Computer)	**Knowledge:** 14 MCQ and 4 free text questions<br>**Attitude:** scale (1–6)<br>**Satisfaction:** evaluation form, scale (1–6)
McDonough 2002	RCT	UK	eLearning vs. traditional learning	37	Third year	Medicine	**CG:** after pre-testing and 20-minute preliminary lecture, students received 90 minutes of face-to-face tutorial in small group. Students in the 90-minute tutorial groups (each n=8 or less) worked with MMD through the same 4 questions in an interactive way.<br>**IG:** students in the FearFighter condition worked alone for 90 minutes exploring the system for instructions on how to answer these 4 questions	• 90 minutes<br>• the abridged HTML version of FearFighter; software installed onto 20 PCs	**Knowledge:** MCQ<br>**Satisfaction:** rating scale
McMullan 2011 [a]	cRCT	UK	eLearning vs. traditional learning	48	Second year	Nursing	**CG:** students were assigned to traditional handout learning support<br>**IG:** students were assigned to non interactive, self-contained, internet independent e-learning PDF drug calculations package, based on cognitive load theory	• time N/A, self directed over 12 weeks<br>• e-learning PDF drug calculations package	**Knowledge:** 20 questions covering the main types of drug calculations<br>**Attitude:** the drug calculation self-efficacy scale<br>**Satisfaction:** a support material satisfaction scale
McMullan 2011 [b]	cRCT	UK	eLearning vs. traditional learning	50	Second year	Nursing	**CG:** students were assigned to traditional handout learning support<br>**IG:** students were assigned to an interactive, self-contained, internet independent e-learning PDF drug calculations package, based on cognitive load theory	• time N/A (self directed over 12 weeks)<br>• e-learning PDF drug calculations package	**Knowledge:** 20 questions covering the main types of drug calculations<br>**Attitude:** the drug calculation self-efficacy scale<br>**Satisfaction:** a support material satisfaction scale
Miedzybrodzka 2001	RCT	UK	eLearning vs. traditional learning	48	Fourth year	Medicine	**CG:** students were assigned to conventional lectures<br>**IG:** students were assigned to CAL package with technical (but not academic) support available, using interactive Model Patient approach in which the student is led through an at-risk patient's process of care from presentation to the general practitioner to consultations at the genetic clinic and screening options available	• control group 20 minutes, intervention group on average 16.4 min<br>• interactive multimedia CAL package, Model Patient application via computer	**Knowledge:** essays and MCQ<br>**Attitude:** Likert scale
Morgulis 2012	RCT	Australia	eLearning vs. eLearning	42	Sixth year	Medicine	**CG:** students were provided with links and encouraged to utilise currently available e-learning resources on leukaemia<br>**IG:** students were assigned to a newly built module	• 2 weeks<br>• computer, internet access	**Knowledge:** single best answer MCQ, multiple response MCQ, drag-and-drop<br>**Attitude:** Likert scale, free text response
Nance 2009	RCT	USA	eLearning vs. traditional learning	73	First year	Dentistry	**CG:** students had access to the carving lab and were given instructional handouts<br>**IG:** DVD only group, students did not receive handouts or attended labs	• time N/A (1 hour for the intervention group)<br>• CAI instructional DVD	**Skills:** grading rubric by experts and students, competency exam<br>**Attitude:** survey

STUDY ID	Methods			Participants			Interventions		
	STUDY DESIGN	LOCATION	COMPARISON	TOTAL NUMBER	YEAR OF STUDY	HEALTHCARE SPECIALITY	CHARACTERISTICS	TIME AND TECHNOLOGY	TEST / OUTCOMES
Nola 2005	RCT	Croatia	eLearning vs. traditional learning	85	Sixth year	Medicine	**CG:** students attended lectures and microscopy sessions with seminars<br>**IG:** students could attend lectures, seminars were substituted by computer with stored pictorial teaching material (photographs, legends for each chapter, list of keywords with explanation, clinical cases with questions and discussion and review questions in the end of each chapter)	• 1 academic year<br>• computers, program with pictorial teaching material	**Knowledge:** exam
Nousiainen 2008	RCT	Canada	eLearning vs eLearning	24	First and second year	Medicine	All participants initially underwent a 7-minute training session<br>**CG:** 6-phase version of the video, watched only once<br>**IG I:** students were able to access video in a self directed manner between and during practice attempts<br>**IG II:** students were able to use the video in the same way as students in intervention I during the first 9 practice sutures; they then had expert instruction (4:1 student to faculty ratio) on suture and knot-tying technique prior to completing the final 9 practice attempts	• 30–40 minutes<br>• interactive video via computer	**Skills:** Imperial College Surgical Assessment Device and global rating scale by 2 blinded experts
Perfeito 2008	RCT	Brazil	eLearning vs. traditional learning	35	Fourth year	Medicine	**CG:** students were assigned to the lecture<br>**IG:** students were assigned to the independent study with a designed program	• 1.5 hour<br>• CD-ROM, computer	**Knowledge:** MCQ and descriptive questions
Prinz 2005	RCT	Switzerland	eLearning vs. eLearning	172	N/A	Medicine	**CG:** students could see surgeon's view (video) of the cataract and glaucoma procedure<br>**IG:** students could see the director's cut of the same procedures, which includes the 3D animations in addition to the surgeon's view sequences identical to those in the control group; both groups had the same narrated comments	• 20 minutes<br>• videos, 3D animations, DVD, storyboard, professional software The presentations were presented over a PC beamer in the same lecture theatre	**Knowledge:** MCQ<br>**Attitude:** questionnaire with 4 level ordinal scale<br>**Satisfaction:** questionnaire with 4 level ordinal scale
Pusic 2007	RCT	Canada	eLearning vs eLearning	139	Final year	Medicine	**CG:** students used linear (PowerPoint®) computer tutorial<br>**IG:** student used branched version (web based) of a computer tutorial	• 2 hours<br>• PC, internet access	**Knowledge:** improvement in the ability to correctly classify 10 CSXRs<br>**Satisfaction:** Likert scale
Qayumi 2004	RCT	Japan	eLearning vs. traditional learning	99	Sixth year	Medicine	**CG:** no intervention<br>**IG I:** students used text module<br>**IG II:** Students used CyberPatient program<br>**IG III:** Students used both, text module and CyberPatient program	• 4 hours<br>• computer	**Knowledge:** MCQ<br>**Skills:** OSCE
Roppolo 2011	RCT	USA	eLearning vs. traditional learning	180	First year	Medicine	**CG:** students attended traditional course with manikin, 4–5 hours in duration<br>**IG I:** students used HeartCode BLS System: web-based, self directed, self paced program for cognitive part<br>**IG II:** students used BLS anytime for healthcare professionals program	• traditional course 4–5 hours, HeartCode 2 hours, BLS aytime 2–2.5 hours<br>• HeartCode BLS system, BLS Anytime system and LaerdalTM Resusci Annie voice activated manikin (VAM) system, HeartCode BLS system	**Skills:** adult CPR skills checklist

STUDY ID	Methods			Participants			Interventions		
	STUDY DESIGN	LOCATION	COMPARISON	TOTAL NUMBER	YEAR OF STUDY	HEALTHCARE SPECIALITY	CHARACTERISTICS	TIME AND TECHNOLOGY	TEST / OUTCOMES
Seabra 2004	RCT	Brazil	eLearning vs. traditional learning	60	Second and third year	Medicine	**CG:** students were assigned to the lecture on epidemiology, diagnosis, clinical manifestation and treatment of prostate cancer<br>**IG:** students were assigned to the multimedia program	• 2 hours<br>• computer, multimedia program	**Knowledge:** 25 MCQ
Shomaker 2002	RCT	USA	eLearning vs. traditional learning	94	Second year	Medicine	**CG:** students attended lectures and were permitted to use syllabus notes, outside texts and a 35-mm slide collection<br>**IG I:** students were assigned to parasitology computer program, syllabus notes and outside texts<br>**IG II:** the combined group had access to all course material	• time N/A<br>• computer, parasitology computer program	**Knowledge:** 42 MCQ and 25 slides<br>**Satisfaction:** a comprehensive course evaluation
Solomon 2004	RCT	USA	eLearning vs. traditional learning	29	Third year	Medicine	**CG:** students travelled to the host community campus and attended the live lectures with their colleagues who chose not to participate in the study<br>**IG:** students stayed at their home campus on the same day and completed a parallel set of CD-ROM-based multimedia modules made from digital recordings of the previous year's lectures. They completed these digital lectures in computer laboratories in either the community campus office or within one of the teaching hospitals	• 6 lectures<br>• CD-ROM based multimedia modules	**Knowledge:** examination (4–5 questions)
Tunuguntla 2008	RCT	USA	eLearning vs. eLearning	49	First year	Medicine	**CG:** students used interactive online model that depicted common home safety issues in static graphs<br>**IG:** students used interactive online model that depicted common home safety issues in animations	• time N/A<br>• computer, online model	**Knowledge:** competency assesment test
Vichitvejpaisal 2001	RCT	Thailand	eLearning vs. traditional learning	80	Third year	Medicine	**CG:** students spent their time reading 275-page textbook<br>**IG:** students were given access to a room which was well equipped with computers and where a 455-electronic page software program was available for each one (CAI)	• 10 hours<br>• computer, CAI program	**Knowledge:** 30-item type K examination
Vivekananda-Schmidt 2005 a*	cRCT	UK	eLearning vs. traditional learning	105	Third year	Medicine	**London:** students allocated to the intervention were given a verbal introduction to the content of the CD-ROM, and each student was given a CD	• 1 day<br>• CD-ROM, computer, video	**Skills:** OSCE<br>**Attitude:** 15 item confidence log
Vivekananda-Schmidt 2005 b*	cRCT	UK	eLearning vs. traditional learning	156	Third year	Medicine	**Newcastle:** students allocated to the intervention arm were each given a CD followed by 1 hour access time to a computer laboratory during lunchtime (arranged in response to findings of the pilot, which suggested that CD use would be higher if access to computers was better)	• 1 day<br>• CD-ROM, computer, video	**Skills:** OSCE<br>**Attitude:** 15 item confidence log

	Methods			Participants			Interventions		
STUDY ID	STUDY DESIGN	LOCATION	COMPARISON	TOTAL NUMBER	YEAR OF STUDY	HEALTHCARE SPECIALITY	CHARACTERSTICS	TIME AND TECHNOLOGY	TEST / OUTCOMES
Weih 2008	RCT	Germany	eLearning vs. traditional learning	101	Eighth semester	Medicine, psychology	**CG:** traditional lecture<br>**IG:** CD-ROM, interactive LMS (LearnCube) in addition to traditional lecture	• traditional lecture 2 times 45 minutes, 1.3 hours for the programme<br>• home PC, Pentium II MS Windows 98 SE, LearnCube-Software	**Knowledge:** 20 MCQ<br>**Satisfaction:** 20 item questionnaire
Williams 2001	RCT	UK	eLearning vs. traditional learning	163	Fourth year	Medicine	**CG:** lecture with videotape, covering a detailed history of the presenting complaint and a mental state examination<br>**IG:** excerpts from the same video were used in the computer-based package. Video clips within the package show the referrer assessing the presenting problem and the patient describing their own symptoms, and provide key background information. Students are prompted to seek further information by carrying out physical, social and psychological investigations, and further video clips allow the learner to carry out a structured mental state examination before making a differential diagnosis. Users of the program can go at their own pace (although in this study, time was limited to match that available for students receiving the lecture) and navigate their own way through the package. Learners get regular feedback on the decisions they are making and are able to test their knowledge and skills in recognising mental state phenomena	• 55 minutes<br>• computer based package, computer	**Knowledge:** 10 5-stem MCQ and mental state exam
Xeroulis 2007	RCT	Canada	eLearning vs. traditional learning	60	First year	Medicine	**CG:** no additional intervention (control)<br>**IG I:** self-study with computer based video instructions, students could interact with the program in continuous replay and slow motion replay modules, watch the skills in their entirety, or access only sections specific to their learning needs<br>**IG II:** expert feedback during practice trials (concurrent feedback)<br>**IG III:** expert feedback after practice trials (summary feedback)	• 1 hour<br>• video instruction tool	**Skills:** global rating scale and Imperial College Surgical Assessment Device

CG = Control group
IG = Intervention group

*Publication contained two studies

5: Risk of bias in included studies

Risk of bias for the 41 parallel RCTs will be covered in this subsection and risk of bias for the eight cluster RCTs will be presented in the subsection thereafter.

Overall the majority of the included parallel RCTs were considered to be of low quality because of high risk of bias.[31,34–37,39,41–47,50,53–55,59,60,65,66,69–72,74] Only a few studies[30,40,49,51,52,56,58,61–64,68,69,75,76] were of high quality with none of the assessed categories rated as high risk of bias (Figure 3). The majority of studies had one or more categories classified as unclear risk of bias, especially with regards to the allocation of participants to intervention groups - see Figure 3 (Risk of bias graph) and Figure 4 (Risk of bias for each individual parallel RCT separately).

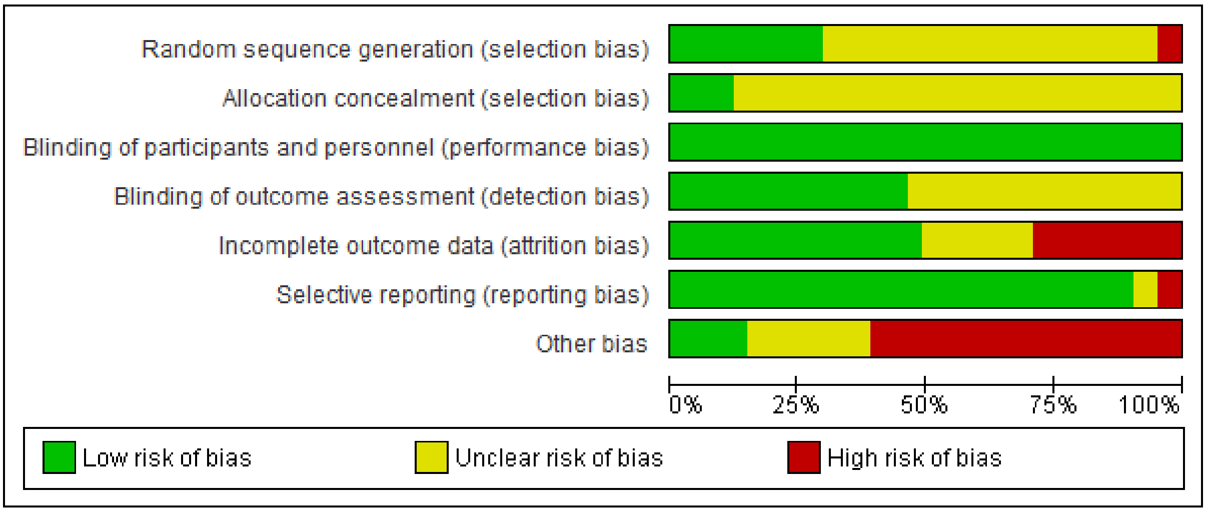

Figure 3: Risk of bias graph

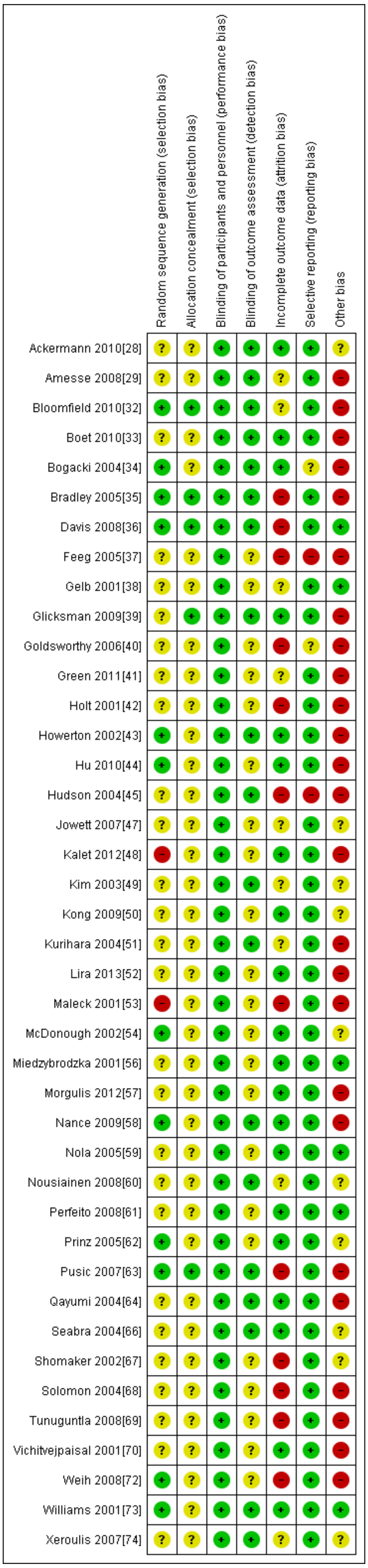

Figure 4: Risk of bias for each individual parallel RCT separately

Random sequence generation and allocation (selection bias)

Most studies (27 of the 41 studies, 66%) included little or no information about the *random sequence generation* and were therefore classified as having an unclear risk of bias.[30,31,35,39–44,47,49,51–54,58,59,61–63,66,68–72,76] Of the remaining studies, only two[50,55] had a high risk of bias for *Random sequence generation*. One[50] of these studies generated the allocation sequence by assigning students to an intervention in the order with which they were entering the room. The other study[55] classified as high risk used radioactive decay numbers to generate the random sequence. Although this is considered a good method, the investigators did not randomise all participants in this way as 20 students were allocated to the control group for practical reasons. The *Random sequence generation* was judged to result in a low risk of bias for 12[34,36–38,45,46,56,60,64,65,74,75] of the 41 studies (29%).

The method used in the majority of the cases to generate a random number sequence was computer software[34,36,38,45,46,60,65,74,75]. Furthermore, two studies used a random number table[37,64] and one[56] used “odd” and “even” conditions from a random number series.

There was no information about the *allocation concealment* method in 36[30,31,35,36,39,40,42–47,49–56,58–64,66,68–72,74–76] out of the 41 trials (88%) and therefore these studies were classified as having an unclear risk of allocation bias. Five studies (12%)[34,37,38,41,65] had a low risk of allocation bias. Two[34,65] of the five studies classified as low risk of bias generated the random numbers on a computer and the numbers were delivered in a way that ensured concealment of allocation, whereas the remaining three studies[37,38,41] all used opaque envelopes for concealment.

Blinding (performance bias and detection bias)

The risk of bias assessment for *blinding of participants and personnel* focused only on the knowledge and skills outcomes. The risk of bias was classified as low for all studies, even though blinding of participants and personnel was not possible in any of these studies because of the nature of the interventions. Our assessment was based on the fact that the 35 studies[30,31,34,36–40,42–47,50–56,58,59,61,63–66,68–72,74,75] investigating knowledge and the six studies[35,41,49,60,62,76] where only skills were measured had an objective outcome assessment. Therefore, the assessment was considered impervious to the student’s opinion about the teaching method. As indicated before, subjective outcomes such as attitudes and student satisfaction were not included in the risk of bias assessment for *blinding of participants and personnel*. Subjective outcomes are more prone to performance bias when participants aren’t blinded due to the fact that the participants’ responses are easily affected by e.g. concerns of consequences of responding negatively to a program developed by the lecturer. Attitudes and student satisfaction would therefore have resulted in a high risk of bias in all of the included studies.

Nineteen[30,31,34–38,41,45,47,51,53,60,62,65,66,68,75,76] of the 41 RCTs (46%) were considered to be at low risk of bias for the *blinding of outcome assessment.* The risk of bias was not only considered low risk in studies where all outcome assessors were blinded but also in studies with unblinded assessors if the method of outcome assessment included no element of interpretation and a classification of a result could be done unambiguously e.g. only assessment was a multiple choice test. The remaining 22 studies[39,40,42–44,46,49,50,52,54–56,58,59,61,63,64,69–72,74] (54%) were rated as having an unclear risk of bias due to lack of information about the blinding of the outcome assessors.

Incomplete outcome data (attrition bias)

As a consequence of the fact that none of the students were blinded there is a high risk of attrition bias for any outcome that relies on active participation of students for follow-up (e.g. answering a questionnaire on attitudes and satisfaction and taking a knowledge test).

A substantial number (12 out of 41, 29%) of the studies[37–39,42,44,47,55,65,69–71,74] did not report complete outcome data (e.g. only reported the mean test score but did not report the number of students who were analysed) or had differential drop-out rates in the different intervention groups and were consequently classified as high risk of bias. Seven of the studies classified as high risk of bias studies[38,39,44,65,69–71] showed a difference in the attrition/exclusion rates between the intervention groups. Five studies[37,42,47,55,74] that were classified as having a high risk of bias had missing/unreported data and did not account for or comment on this.

Twenty (49%) studies[30,35,36,41,45,46,50,52,54,56,58–61,63,64,66,68,72,75] were classified as low risk of bias for *incomplete outcome data.* These studies reported if attrition and exclusion had occurred. The information provided regarding the reason for not analysing all participants was either similar for the groups being compared and/or showed only a small and statistically insignificant difference between the studies.

Because details of attrition and exclusion were not reported, nine studies (22%)[31,34,40,43,49,51,53,62,76] were classified as unclear risk of bias for *incomplete outcome data*. In these studies it was not clear if there was excessive drop-out in one group compared to the other(s) or if it had occurred at all.

Selective reporting (reporting bias)

The majority of studies (37 out of 41, 90%)[30,31,34,35,37,38,40,41,43–46,49–56,58–66,68–72,74–76] were rated as low risk of *selective reporting bias*. This was mainly due to the categorization criteria for low risk of bias that only required the authors to report results for all outcomes reported in the methods sections of the published articles; protocols were not available to our reviewers. Only two studies[36,42] were rated as having an unclear risk of bias (5%). This was a result of the authors not presenting sufficient details on planned tests to allow us to assess the risk of selective reporting bias. Similarly, only two[39,47] out of the 41 studies (5%) were categorized as having a high risk of selective reporting bias. One of these studies[47] described one or more outcome measures that they had investigated and then did not report them in the results. The other study[39] omitted two questions out of 20 in the analysis of the results without giving any explanation for the exclusion or results for them, and only reported the comparison between the controls and a subgroup of the intervention group rather than the entire intervention group.

Other potential sources of bias

Volunteer bias is an important and sometimes almost inevitable problem in studies assessing different ways of learning. Volunteer bias therefore resulted in a high risk of bias classification in 18 of the 41 included studies (44%).[31,34,36,37,41,44–47,50,59,60,65,66,70–72,74] It was unclear whether volunteer bias was a problem in 14 of the 41 studies[30,35,39,42,43,49,51,53,54,56,62,64,68,76] (34%). Only nine studies (22%) randomized entire class rooms or the entire year, and were therefore at low risk of volunteer bias.[38,40,52,55,58,61,63,69,75]

We classified nine studies (22%)[35,39,42,43,47,54,55,66,74] as having a high risk of bias other than volunteer bias and types earlier described. Five of these studies[35,39,42,43,54] suffered from imbalanced comparison groups where more material or information was given in one group compared to others. This was only the case for the intervention group and thus biased the results away from the null. Contamination (i.e. the control group was also exposed to the eLearning intervention) was also a problem and concern in one study[74] that was categorised as high risk of bias. However, it is possible that contamination occurred in several of the other included trials as it is likely that students shared material with course mates who were randomised to a different group. A study investigating different computer-based formats[47] had differential attendance among the different eLearning interventions and was therefore also considered to be at high risk of bias. Another study[55] categorised as having a high risk of bias was the study that breached the RCT design because 20 students were added to the control group without having been randomised as such; the analysis of results did not take this into account following a per protocol analysis rather than intention to treat. Finally, one study of academic performance of medical students[53] only presented some of the results stratified by the different intervention groups whereas the rest were presented stratified by performance groups; thus not all analyses are reported according to the group they were randomised to.

Seven studies[35,37,45,46,49,51,69] (17%) were classified as having an unclear risk of other bias. Three of these studies[37,51,69] had (either) 1-2 students attending interventions they were not allocated to or the reviewer was unable to assess whether contamination could have taken place. One study[46] had small baseline differences that were likely to have occurred by chance. Another study[35] failed to report any information on who the students recruited were (i.e. course, year etc.). A study of teaching methods for intraoral radiography[45] did not clearly state what the control group was exposed to. Finally, a study investigating teaching methods for surgical skills [49] did not compare two different intervention methods, but instead exposed one group to longer time with the intervention.

Due to several types of bias being assessed under *other potential sources of bias* we classified *other bias* as high risk of bias if one of the elements assessed was of high risk even though other elements were unclear or low. For example if there was a high risk of volunteer bias, but a unclear risk of contamination we would classify it as having a high risk of Bias. Please refer to Figure 3 for the assessment per study.

Risk of bias in cluster RCTs

Eight studies reported in six articles included in our review were cluster RCTs.[32,33,48,57,67,73] The methods and analyses employed in these cluster RCTs were generally not judged to be of high quality due to one or more risk of bias items being categorised as high risk of bias.

The recruitment process and recruitment bias was not addressed in six[32,33,48,57,67] of the eight included studies. The remaining two studies[73] that were judged to be of low risk of recruitment bias had provided enough information on the participant flow and randomization process for this assessment to be made.

Baseline characteristics differed between the intervention and control group in six studies.[33,57,67,73] In two studies,[57] the authors chose not to combine the results of two separate cluster RCTs because of these differences. In the other four studies[33,67,73] there was a difference in previous experience with the field being taught or experience in

using a computer between the intervention and control group. These studies were therefore all judged to be of high risk of bias affecting the outcome. Two studies[32,48] provided no information on baseline characteristics and whether these were different between the groups.

None of the studies reported loss of entire clusters, however, all but one study[32] reported drop-out of individual participants. Six[33,57,67,73] of the studies had a high drop-out rate that resulted in a high-risk of bias classification. One study investigating eLearning as a method of teaching skills for performing electrocardiographs (ECGs)[48] reported attrition, but this study was judged to have a low risk of bias because the attrition was limited and was very unlikely to have affected the results.

Two studies examining methods of teaching musculoskeletal examination skills[73] accounted for the cluster unit in the analysis of the results. The rest of the cluster RCT studies[32,33,48,57,67] suffered from unit of analysis error (i.e. incorrectly analysed participants as independent individuals rather than the unit they were randomized in)[26]. Therefore, in these studies there is a high risk of false positive conclusions. Two studies of teaching methods for drug calculation skills[57] addressed the issue of a reduced effective sample size due to the nature of the cluster RCT design but did not account for it in the data analysis.

Volunteer bias was only a problem in one of the cluster RCTs.[67] In another study[32] it was unclear whether or not there was a risk of volunteer bias. The remaining six studies[33,48,57,73] were all categorised as having a low risk of volunteer bias.

In the study by Roppolo et al.[67]there was a high risk of selective outcome reporting because the authors state that cognitive testing took place but did not report the results.
